# The Absence of Calponin 2 in Rabbits Suggests Caution in Choosing Animal Models

**DOI:** 10.3389/fbioe.2020.00042

**Published:** 2020-02-28

**Authors:** Olesya Plazyo, Weilong Hao, Jian-Ping Jin

**Affiliations:** ^1^Department of Physiology, School of Medicine, Wayne State University, Detroit, MI, United States; ^2^Department of Biological Sciences, Wayne State University, Detroit, MI, United States

**Keywords:** CRISPR/Cas9 gene editing, rabbit, comparative genomics, protein profiling, calponin 2

## Abstract

While the rapid development of CRISPR/CAS9 technology has allowed for readily performing site-specific genomic editing in non-rodent species, an emerging challenge is to select the most suitable species to generate animal models for the study of human biology and diseases. Improving CRISPR/CAS9 methodology for more effective and precise editing in the rabbit genome to replicate human disease is an active area of biomedical research. Although rabbits are more closely related to humans than mice (based on DNA sequence analysis), our whole-genome protein database search revealed that rabbits have more missing human protein sequences than mice. Hence, precisely replicating human diseases in rabbits requires further consideration, especially in studies involving essential functions of the missing proteins. For example, rabbits lack calponin 2, an actin-associated cytoskeletal protein that is important in the pathogenesis of inflammatory arthritis, atherosclerosis, and calcific aortic valve disease. The justification of using rabbits as models for human biomedical research is based on their larger size and their closer phylogenetic distance to humans (based on sequence similarity of conserved genes), but this may be misleading. Our findings, which consider whole-genome protein profiling together with actual protein expressions, serve as a warning to the scientific community to consider overall conservation as well as the conservation of specific proteins when choosing an animal model to study a particular aspect of human biology prior to investing in genetic engineering.

## Introduction

The rapid development of CRISPR/Cas9 technology for readily editing mammalian genomes has allowed for the efficient generation of genetically engineered animal models for the study of human diseases in non-rodent species (Liu et al., 2018). The rabbit, in particular, has become a commonly used animal model for the study of molecular mechanisms underlying human biology and disease (Esteves et al., 2018). Historically, rabbits have been utilized for the development of vaccines (starting with Louis Pasteur’s rabies vaccine in 1881) (Pasteur, 1885), production and characterization of antibodies (Weber et al., 2017), early molecular immunology (Esteves et al., 2018), and experimental surgery (Calasans-Maia et al., 2009), among other applications, due to advantages such as strong immune response to foreign antigens, greater availability of cells and tissues from a single animal, and ease of manipulation because of the larger size. Rabbits are considered an intermediate between broadly studied rodents and larger but costlier mammals. In recent years, using CRISPR/Cas9 technology, genetically modified rabbits have been promoted in studies of human diseases ranging from muscular dystrophy (Sui et al., 2018) and hypertrophy (Lv et al., 2016) to retinal degeneration (Kondo et al., 2009), atherosclerosis (Wang et al., 2013), and X-linked hypophosphatemia (Sui et al., 2016). Intrigued by the increasing power and efficiency to generate mutant rabbits for models of human diseases and the rich historical literature of rabbit research on immunological (Esteves et al., 2018) and cardiovascular disorders (Hasenfuss, 1998), we explored this approach for a potential expansion of our research on calponin, a family of regulators of actin cytoskeleton, that plays a role in the regulation of actin cytoskeleton-mediated cell motility (Liu and Jin, 2016a).

**FIGURE 1:**
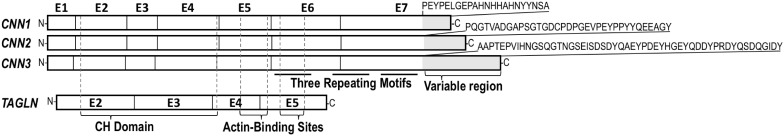
Human calponin isoforms and transgelin. *CNN1* (GenBank NC_000019.10, region 11538775.11550323), *CNN2* (GenBank NC_000019.10, region 1026608.1039065), and *CNN3* (GenBank NC_000001.11, region complement 94896949.94927223) genes each consists of seven exons. The three calponin isoforms are conserved in structure except the C-terminal region that is diverged in length and amino acid sequences as shown in the figure. *TAGLN* (SM22alpha) (GenBank NC_000011.10, region 117199294.117207465) contains only four coding exons corresponding to the conserved core structure of calponin family. Positions of the CH (calponin homology) domain and two actin binding sites are indicated in the alignment map.

Three homologous genes (*CNN1*, *CNN2*, and *CNN3*) have evolved in vertebrates to encode three isoforms of calponin (calponin 1, 2, and 3). Each of the calponin isoform genes is composed of seven exons with a variable region at the C-terminus that differentiates the isoforms (Figure 1). Although the calponin isoforms have significantly diverged during evolution, each isoform is well conserved in the vertebrate phylum as has been shown for numerous species, including channel catfish, Western clawed frogs, brown tree snakes, black flying foxes, chickens, mice, hamsters, chimpanzees, and humans, among others (Liu and Jin, 2016a). The expression and function of each calponin isoform are distinct. Calponin 1 is specifically expressed in fully differentiated smooth muscle cells and contributes to regulating contractility (Takahashi et al., 2000; Feng et al., 2019). Calponin 2 is expressed in a broader range of cell types, including fibroblasts, macrophages, cancer cells, and others to participate in regulating cellular responses to mechanical signals, cell proliferation, and motility (Qiu et al., 2017; Plazyo et al., 2018, 2019). Calponin 3 is the least studied of the three isoforms. It is expressed in the brain with a potential contribution to neural plasticity (Ferhat et al., 2003) and in placenta where it mediates trophoblastic cell fusion (Shibukawa et al., 2010).

Extensive research in the past three decades demonstrates that each of the calponin isoforms have evolved with discrete functions and expression regulation as observed across a variety of vertebrate species (Liu and Jin, 2016a). The present study provides the first report that the rabbit is a unique species that lacks calponin 2, a cytoskeleton regulatory protein conserved in all other vertebrates studied to date. Using Western blotting to examine actual protein expression and whole-genome comparison to reveal the difference between humans and rabbits versus humans and mice, our data present a striking example of the loss of a gene with important functions, suggesting that caution should be used when selecting rabbits as animal models to study human biology and disease.

## Results

**FIGURE 2:**
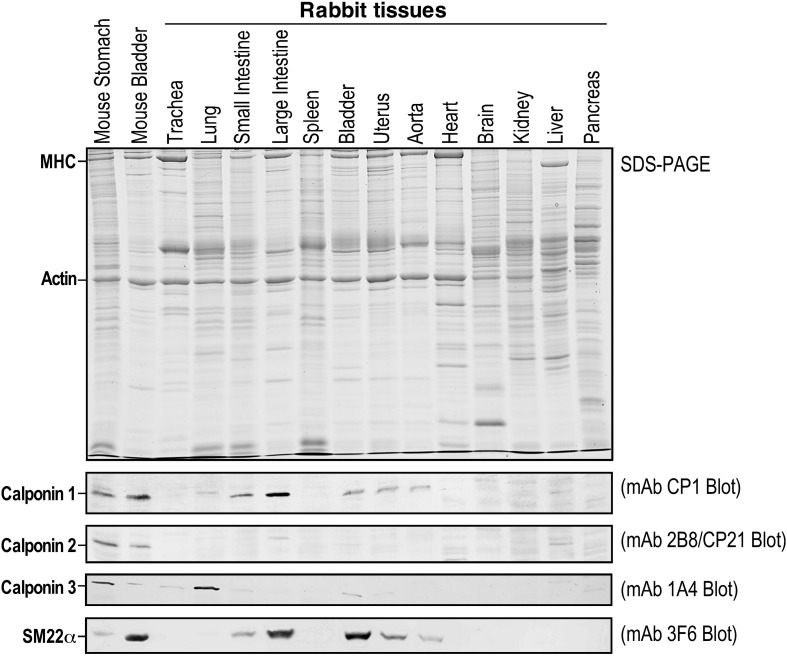
Calponin 2 is absent in rabbits. Rabbit tissue lysates were subjected to SDS-PAGE (top panel) and immunoblotting using monoclonal antibodies (mAbs) specific to calponin 1 (CP1) (Jin et al., 1996), calponin 2 (2B8/CP21) (Hossain et al., 2006), calponin 3 (1A4 raised against mouse calponin 3 expressed from cloned cDNA) or SM22alpha/transgelin (3F6) (Liu et al., 2017) (lower panels). While calponin 1 and calponin 2 have similar mobility in the SDS-gel used for Western blotting, calponin 3 and SM22alpha are readily distinguished from calponin 1 and calponin 2 by their molecular weights. Mouse stomach and bladder were used as positive controls. Whereas the expression of calponin 1, calponin 3, and SM22alpha was detected in specific tissue types as previously shown in mice and humans, calponin 2 is completely absent in rabbit tissues as demonstrated in the mAb 2B8/CP21 blot. MHC, myosin heavy chain.

Considering that the three calponin isoforms are conserved in all vertebrate species studied in the past, it was surprising that our initial examination failed to detect the calponin 2 protein in all of the rabbit tissues that are known to express calponin 2 in humans and mice. Using specific, previously characterized monoclonal antibodies (Hossain et al., 2006), we assessed the expression of all calponin isoforms in total protein extracts from the trachea, lungs, small intestine, large intestine, spleen, bladder, uterus, aorta, heart, brain, kidney, liver, and pancreas by Western blot. Figure 2 demonstrates that only calponin 1 and calponin 3 were detected in the rabbit, whereas the cell motility regulator, calponin 2, was not found. We verified the results by multiple immunological examinations using several monoclonal and polyclonal antibodies against calponin 2 with confirmed cross-species reactivity (Jin et al., 1996) (data not shown).

We then searched the Ensembl and NCBI sequence databases and found no entry for rabbit calponin 1 and calponin 2, confirming the lack of calponin 2 as originally observed by Western blot. However, the calponin 1 protein is detected at significant levels in rabbit smooth muscles using Western blotting (Figure 2). To verify the initial search results, human *CNN1*, *CNN2*, and *CNN3* isoforms were used as query sequences in BLAST searches against mouse and rabbit protein databases. To investigate the presence of the calponin genes in mice and rabbits, we collected proteins with a BLASTP *E*-value less than 10^–30^. Protein sequences were aligned using MUSCLE (Edgar, 2004). A maximum likelihood tree was constructed using PhyML (Guindon et al., 2010). The results found only one calponin (*CNN3*) in the rabbit. This was further confirmed by TBLASTN searches using human *CNN1*, *CNN2*, and *CNN3* as query sequences, all of them match the same genomic region and *CNN3* has the highest similarity. Therefore, the presence of the apparently functional *CNN1* gene in the rabbit was not detectable by using the common sequence database searches, demonstrating the critical value of direct detections at the protein level (Figure 2). We also performed a HMMER search at https://www.ebi.ac.uk/Tools/hmmer/search/phmmer (Potter et al., 2018). When the query search is with the human *CNN2* protein, then the top HMMER hit is calponin 3 in rabbit and the second best hit is to transgelin. These HMMER results confirm the BLASTP results.

To obtain a comprehensive understanding of this unexpected finding, we also performed systemic BLASTP homology searches of all human protein genes against mouse and rabbit genomes with *E*-values < 10^–5^. The BLASTP search revealed the absence of 843 distinct human protein coding genes in rabbit proteome (Table 1), among which many are critical to essential functions such as immune regulation, cell cycle progression, mitochondrial energetics, transcription/chromatin remodeling, lipid metabolism, ER/endosome trafficking, calcium signaling, neuropeptides, and spermatogenesis (Supplementary Table S1). In addition, gene ontology (GO) analysis detected 157 human genes missing in the rabbit genome (Supplementary Figure S1).

**TABLE 1:** Rabbits have fewer human homologs than mice.

	Human protein sequences	% Human sequences	Human protein isoforms	% Human isoforms	Human protein Names	% Human protein names
Absent in Rabbit	2,146	1.89%	1,475	2.09%	843	4.23%
Absent in Mouse	1,454	1.28%	1,019	1.44%	652	3.27%
% Absent in Mouse/Absent in Rabbit	67.8%		69.1%		77.3%	
Unique to Rabbit	284	0.25%	211	0.30%	83	0.42%
Unique to Mouse	978	0.86%	667	0.94%	274	1.38%
% Unique to Rabbit/Unique to Mouse	29.0%		31.6%		30.3%	

*A comparative genomic analysis was performed to determine how many unique human protein sequences, unique human protein isoforms, or unique human protein names were missing in the database for rabbits or mice. An additional analysis was performed to identify which proteins were present in rabbits but not in mice (unique to rabbit) or in mice but not rabbits (unique to mouse).*

Mice have been extensively used to develop genetically modified models for the research of human diseases. Although the use of mice is continuously criticized as unideal in replicating human physiology and disease (Nature Medicine Editorial, 2013), the same comparison of human and mouse genomes found an absence of a significantly smaller number (652) of distinct human protein coding genes in mice (Table 1). Both rabbits and mice belong to the *Glires* clade with the mouse lineage having a longer branch length than the rabbit lineage based on the sequence similarity of conserved genes (Murphy et al., 2001). However, the sequence alignment approach to classification may be misleading, as whole-genome protein profiling appears to be more informative. While rabbits are considered phylogenetically closer to humans than mice based on DNA sequence similarities, the fact that mice have a profile of protein coding genes that are more similar to that of humans than that of rabbits to humans raises concern. To quantitatively evaluate this potentially high impact finding, genomic comparison results in Table 1 demonstrate that the proportion of unique human protein sequences, protein isoforms, or total protein entries in the databases all showed approximately 30% more missing in rabbits than that missing in mice.

## Discussion

Three homologous calponin isoform genes have evolved in vertebrates (Liu and Jin, 2016a). Our finding of the lack of *CNN2* gene in rabbits (Figure 2) calls for caution in using rabbit models in calponin or actin regulation-related research. While *CNN1* functions in smooth muscle contractility (Yamamura et al., 2007) and *CNN3* is a isoform with unclear functions (Liu and Jin, 2016a), calponin 2 functions in many cell motility-related functions. The loss of calponin 2 in myeloid immune cells in mice has been shown to modulate inflammatory responses (Plazyo et al., 2019), such as attenuating inflammatory arthritis (Huang et al., 2016) and the pathogenesis of atherosclerosis (Liu and Jin, 2016b). *CNN2* knock out in mice also reduces myofibroblast differentiation and the development of calcific aortic valve disease (Plazyo et al., 2018) and slows down platelet adhesion in thrombosis (Hines et al., 2014). Reduced expression of calponin 2 is found in metastatic cancer cells (Moazzem Hossain et al., 2014). Therefore, the lack of *CNN2* in rabbits is anticipated to have multiple pathophysiological effects. Our data, reported here, aims to timely inform the research community of an important precaution in order to avoid the resource-consuming construction of genetically modified rabbit models that may be less effective for many areas of research.

**FIGURE 3:**
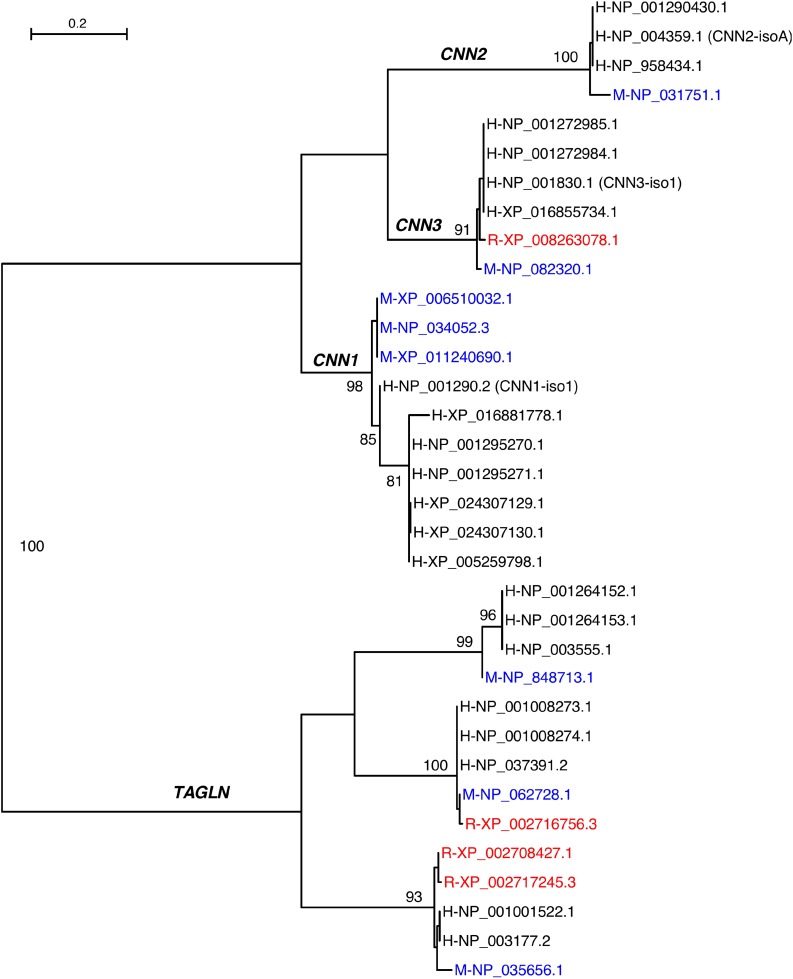
Maximum likelihood phylogeny of calponin and transgelin homologs from humans (H), mice (M), and rabbits (R). Mouse proteins are in blue whereas rabbit proteins are in red. The primary entries of the three human calponin isoforms are indicated in parentheses. The branches are supported by bootstrap values of which values greater than 80% are shown.

The three calponin isoforms have distinct expressions in different cell and tissue types (Figure 2) reflecting specific functions. If co-expressed in the same cell, calponin isoforms may potentially have complementary functions (Feng et al., 2019). Calponins also share high sequence similarities with transgelin (*TAGLN*), also named SM22alpha (Morgan and Gangopadhyay, 2001). Phylogenetic analysis demonstrated that *TAGLN* is clearly related to the calponin gene family (Figure 3). In the genomic search of the rabbit, the second best BLAST hit of each human calponin protein is a transgelin homolog. Although SM22alpha is considerably shorter than calponins 1, 2, and 3, it contains the conserved core structures of calponins (Figure 1). The mechanoregulation of SM22alpha in fibroblasts also resemble that of calponin 2 (Liu et al., 2017), suggesting potentially functional overlap. Therefore, the functional impacts of loss of *CNN2* in rabbit smooth muscles and myofibroblast biology may be partially compensated by calponin 1 and/or SM22alpha (Liu et al., 2017; Feng et al., 2019).

Consistent with the fact that rabbits survive well without the *CNN2* gene, deletion of *CNN2* in mice does not cause lethality (Huang et al., 2008). The absence of calponin 2 in rabbits presents a natural calponin 2-null model that can be employed to study the compensatory functions of calponin 1 and 3 in actin-mediated cellular processes in multiple cell types. In the meantime, comparative genomic analysis can reveal other missing human genes that are critical for the research on particular mechanisms of human diseases to guide targeted humanizations of rabbit and other animal models, for example by using the increasingly powerful CRISPR/Cas9 technology.

The comparative genomics data further showcases that mice actually miss fewer human protein coding genes than that rabbits (Table 1). While the mouse has long been regarded simply as a convenient animal model for advantages such as low cost of housing, relatively short reproductive cycle, large litter size, and ease of genetic manipulations among others, its evaluation should now be revisited for being a more preferable choice over the rabbit due to its higher similarity to humans in genome-wide protein profiles (Supplementary Table S1). Consistent with the substantial divergence of rabbit proteome with that of humans, numerous studies have alluded to the divergence of molecular, cellular, and physiological mechanisms between rabbits and humans.

Improvements in genome editing, such as the generation of point mutations (Liu et al., 2018), will certainly facilitate the production of rabbit models for research, but precautions are imperative to thoroughly consider their precise representation of human conditions. Our example of evaluating animal models of human biology and diseases involving calponin-mediated mechanisms shows that when selecting an animal model to study a specific physiologic mechanism or disease of humans, intuitive preference for larger sized animals may not always translate into closer genetic background that determines the relevance to the research objective, especially in integrative pathophysiological and translational studies.

Finally, it is worth pointing out that, since the differences in whole-genome protein profiles between human and various vertebrates would certainly affect the function of individual proteins *in vivo* and during development, one must consider these differences to understand the often complex phenotype of a particular genetic modification of interest engineered in a particular animal model of human disease. To achieve this goal, it is necessary to reliably detect missing genes in a genome by sensitive homology search analysis. In this present study, we applied multiple homology search methods along with protein level confirmation to reliably demonstrate the lack of a functional *Cnn2* gene in rabbit. This two-level approach – a combination of tests for protein expression and genome search analysis – provides the power to improve our understanding of animal models and their application in biomedical studies.

## Data Availability Statement

All datasets generated for this study are included in the article/Supplementary Material.

## Ethics Statement

The animal study was reviewed and approved by the Wayne State University.

## Author Contributions

OP conducted the experiments, analyzed the data, drafted the manuscript, and approved the submitted version of the manuscript. WH designed and conducted the bioinformatic studies, analyzed the data, drafted the manuscript, and approved the submitted version of the manuscript. J-PJ designed the study, analyzed the data, drafted the manuscript, and approved the submitted version of the manuscript.

## Conflict of Interest

The authors declare that the research was conducted in the absence of any commercial or financial relationships that could be construed as a potential conflict of interest.

## References

[B1] Calasans-Maia M. D. Monteiro M. L. Ascoli F. O. Granjeiro J. M. (2009). The rabbit as an animal model for experimental surgery. *Acta Circ. Bras.* 24 325–328. 10.1590/s0102-86502009000400014 19705034

[B2] Edgar R. C. (2004). MUSCLE: multiple sequence alignment with high accuracy and high throughput. *Nucleic Acids Res.* 32 1792–1797. 10.1093/nar/gkh340 15034147 PMC390337

[B3] Esteves P. J. Abrantes J. Baldauf H. M. BenMohamed L. Chen Y. Christensen N. (2018). The wide utility of rabbits as models of human diseases. *Exp. Mol. Med.* 50:66. 10.1038/s12276-018-0094-1 29789565 PMC5964082

[B4] Feng H. Z. Wang H. Takahashi K. Jin J. P. (2019). Double deletion of calponin 1 and calponin 2 in mice decreases systemic blood pressure with blunted length-tension response of aortic smooth muscle. *J. Mol. Cell Cardiol.* 129 49–57. 10.1016/j.yjmcc.2019.01.026 30707993 PMC6486848

[B5] Ferhat L. Esclapez M. Represa A. Fattoum A. Shirao T. Ben-Ari Y. (2003). Increased levels of acidic calponin during dendritic spine plasticity after pilocarpine-induced seizures. *Hippocampus* 13 845–858. 10.1002/hipo.10136 14620880

[B6] Guindon S. Dufayard J. F. Lefort V. Anisimova M. Hordijk W. Gascuel O. (2010). New algorithms and methods to estimate maximum-likelihood phylogenies: assessing the performance of PhyML 3.0. *Syst. Biol.* 59 307–321. 10.1093/sysbio/syq010 20525638

[B7] Hasenfuss G. (1998). Animal models of human cardiovascular disease, heart failure and hypertrophy. *Cardiovasc. Res.* 39 60–76. 10.1016/s0008-6363(98)00110-2 9764190

[B8] Hines P. C. Gao X. White J. C. D’Agostino A. Jin J. P. (2014). A novel role of h2-calponin in regulating whole blood thrombosis and platelet adhesion during physiologic flow. *Physiol. Rep.* 2:e12228. 10.14814/phy2.12228 25472609 PMC4332209

[B9] Hossain M. M. Smith P. G. Wu K. Jin J. P. (2006). Cytoskeletal tension regulates both expression and degradation of h2-calponin in lung alveolar cells. *Biochemistry* 45 15670–15683. 10.1021/bi061718f 17176089 PMC1764619

[B10] Huang Q. Q. Hossain M. M. Sun W. Xing L. Pope R. M. Jin J. P. (2016). Deletion of calponin 2 in macrophages attenuates the severity of inflammatory arthritis in mice. *Am. J. Physiol. Cell Physiol.* 311 C673–C685. 10.1152/ajpcell.00331.2015 27488671 PMC5129749

[B11] Huang Q. Q. Hossain M. M. Wu K. Parai K. Pope R. M. Jin J. P. (2008). Role of H2-calponin in regulating macrophage motility and phagocytosis. *J. Biol. Chem.* 283 25887–25899. 10.1074/jbc.M801163200 18617524 PMC2533796

[B12] Jin J. P. Walsh M. P. Resek M. E. McMartin G. A. (1996). Expression and epitopic conservation of calponin in different smooth muscles and during development. *Biochem. Cell. Biol.* 74 187–196. 10.1139/o96-019 9213427

[B13] Kondo M. Sakai T. Komeima K. Kurimoto Y. Ueno S. Nishizawa Y. (2009). Generation of a transgenic rabbit model of retinal degeneration. *Invest. Ophthalmol. Vis. Sci.* 50 1371–1377. 10.1167/iovs.08-2863 19074802

[B14] Liu R. Hossain M. M. Chen X. Jin J. P. (2017). Mechanoregulation of SM22alpha/Transgelin. *Biochemistry* 56 5526–5538. 10.1021/acs.biochem.7b00794 28898058

[B15] Liu R. Jin J. P. (2016a). Calponin isoforms CNN1, CNN2 and CNN3: regulators for actin cytoskeleton functions in smooth muscle and non-muscle cells. *Gene* 585 143–153. 10.1016/j.gene.2016.02.040 26970176 PMC5325697

[B16] Liu R. Jin J. P. (2016b). Deletion of calponin 2 in macrophages alters cytoskeleton-based functions and attenuates the development of atherosclerosis. *J. Mol. Cell Cardiol.* 99 87–99. 10.1016/j.yjmcc.2016.08.019 27575021 PMC5325694

[B17] Liu Z. Chen M. Chen S. Deng J. Song Y. Lai L. (2018). Highly efficient RNA-guided base editing in rabbit. *Nat. Commun.* 9:2717. 10.1038/s41467-018-05232-2 30006570 PMC6045575

[B18] Lv Q. Yuan L. Deng J. Chen M. Wang Y. Zeng J. (2016). Efficient generation of myostatin gene mutated rabbit by CRISPR/Cas9. *Sci. Rep.* 6:25029. 10.1038/srep25029 27113799 PMC4844959

[B19] Moazzem Hossain M. Wang X. Bergan R. C. Jin J. P. (2014). Diminished expression of h2-calponin in prostate cancer cells promotes cell proliferation, migration and the dependence of cell adhesion on substrate stiffness. *FEBS Open Biol.* 4 627–636. 10.1016/j.fob.2014.06.003 25161871 PMC4141211

[B20] Morgan K. G Gangopadhyay S. S. (2001). Invited review: cross-bridge regulation by thin filament-associated proteins. *J. Appl. Physiol. (1985)* 91 953–962. 10.1152/jappl.2001.91.2.953 11457814

[B21] Murphy W. J. Eizirik E. Johnson W. E. Zhang Y. P. Ryder O. A. O’Brien S. J. (2001). Molecular phylogenetics and the origins of placental mammals. *Nature* 409 614–618. 10.1038/35054550 11214319

[B22] Nature Medicine Editorial (2013). Of men, not mice. *Nat. Med.* 19:379. 10.1038/nm.3163 23558605

[B23] Pasteur L. (1885). Méthode pour prévenir la rage après morsure. *C. R. T.* 101 765–772.

[B24] Plazyo O. Liu R. Moazzem Hossain M. Jin J. P. (2018). Deletion of calponin 2 attenuates the development of calcific aortic valve disease in ApoE(-/-) mice. *J. Mol. Cell Cardiol.* 121 233–241. 10.1016/j.yjmcc.2018.07.249 30053524

[B25] Plazyo O. Sheng J. J. Jin J. P. (2019). Down-regulation of Calponin 2 contributes to the quiescence of lung macrophages. *Am. J. Physiol. Cell Physiol.* 317 C749–C761. 10.1152/ajpcell.00036.2019 31365293 PMC6850996

[B26] Potter S. C. Luciani A. Eddy S. R. Park Y. Lopez R. Finn R. D. (2018). HMMER web server: 2018 update. *Nucleic Acids Res.* 46 W200–W204. 10.1093/nar/gky448 29905871 PMC6030962

[B27] Qiu Z. Chu Y. Xu B. Wang Q. Jiang M. Li X. (2017). Increased expression of calponin 2 is a positive prognostic factor in pancreatic ductal adenocarcinoma. *Oncotarget* 8 56428–56442. 10.18632/oncotarget.17701 28915602 PMC5593573

[B28] Shibukawa Y. Yamazaki N. Kumasawa K. Daimon E. Tajiri M. Okada Y. (2010). Calponin 3 regulates actin cytoskeleton rearrangement in trophoblastic cell fusion. *Mol. Biol. Cell.* 21 3973–3984. 10.1091/mbc.E10-03-0261 20861310 PMC2982094

[B29] Sui T. Xu L. Lau Y. S. Liu D. Liu T. Gao Y. (2018). Development of muscular dystrophy in a CRISPR-engineered mutant rabbit model with frame-disrupting ANO5 mutations. *Cell Death Dis.* 9:609. 10.1038/s41419-018-0674-y 29789544 PMC5964072

[B30] Sui T. Yuan L. Liu H. Chen M. Deng J. Wang Y. (2016). CRISPR/Cas9-mediated mutation of PHEX in rabbit recapitulates human X-linked hypophosphatemia (XLH). *Hum. Mol. Genet.* 25 2661–2671. 27126636 10.1093/hmg/ddw125

[B31] Takahashi K. Yoshimoto R. Fuchibe K. Fujishige A. Mitsui-Saito M. Hori M. (2000). Regulation of shortening velocity by calponin in intact contracting smooth muscles. *Biochem. Biophys. Res. Commun.* 279 150–157. 10.1006/bbrc.2000.3909 11112431

[B32] Wang Y. Niimi M. Nishijima K. Waqar A. B. Yu Y. Koike T. (2013). Human apolipoprotein A-II protects against diet-induced atherosclerosis in transgenic rabbits. *Arterioscler. Thromb. Vasc. Biol.* 33 224–231. 10.1161/ATVBAHA.112.300445 23241412 PMC3673010

[B33] Weber J. Peng H. Rader C. (2017). From rabbit antibody repertoires to rabbit monoclonal antibodies. *Exp. Mol. Med.* 49:e305. 10.1038/emm.2017.23 28336958 PMC5382564

[B34] Yamamura H. Hirano N. Koyama H. Nishizawa Y. Takahashi K. (2007). Loss of smooth muscle calponin results in impaired blood vessel maturation in the tumor-host microenvironment. *Cancer Sci.* 98 757–763. 10.1111/j.1349-7006.2007.00452.x 17391313 PMC11159921

