# Supplementary figures and images for: The Absence of Calponin 2 in Rabbits Suggests Caution in Choosing Animal Models
Source: Front Bioeng Biotechnol. 2020 Feb 28;8:42. doi: 10.3389/fbioe.2020.00042 (PMC7058930; doi:10.3389/fbioe.2020.00042)

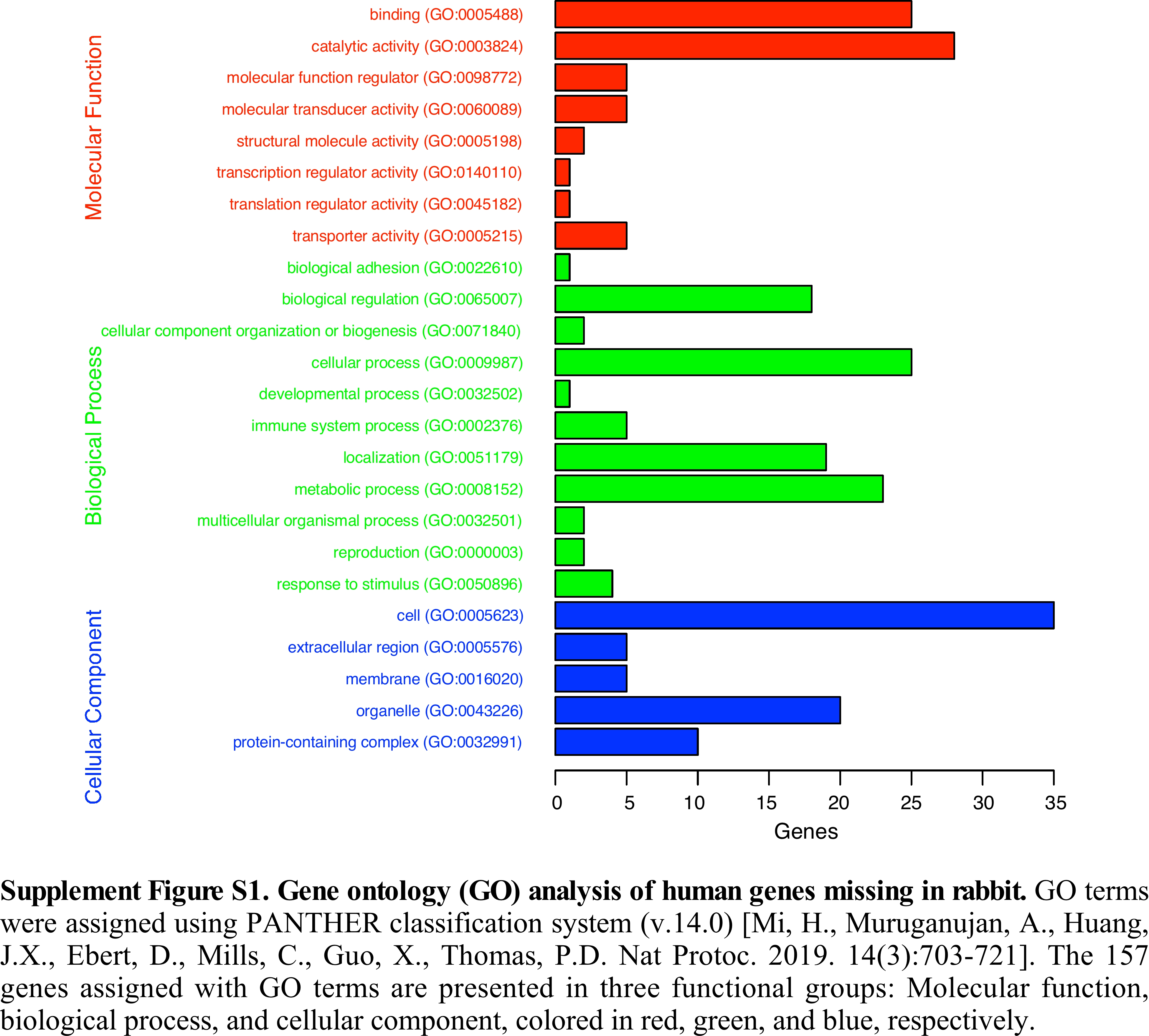

Supplement: Supplementary file 1 [file Image_1.JPEG]
