# Supplementary material for: The Absence of Calponin 2 in Rabbits Suggests Caution in Choosing Animal Models
Source: Front Bioeng Biotechnol. 2020 Feb 28;8:42. doi: 10.3389/fbioe.2020.00042 (PMC7058930; doi:10.3389/fbioe.2020.00042)
Supplement: Supplementary file 2 [file Table_1.pdf]

Supplement Table 1

The unique human protein sequences missing in rabbit and mouse are listed below in alphabetical orders.

Unique human protein sequences missing in rabbit
14-3-3 protein zeta/delta isoform X2
14-3-3 protein zeta/delta isoform X3
28S ribosomal protein S34, mitochondrial isoform 2
28S ribosomal protein S34, mitochondrial isoform X1
36.4 kDa proline-rich protein
36.4 kDa proline-rich protein
39S ribosomal protein L12, mitochondrial
39S ribosomal protein L28, mitochondrial
39S ribosomal protein L28, mitochondrial isoform X1
39S ribosomal protein L28, mitochondrial isoform X1
5-oxoprolinase
acidic fibroblast growth factor intracellular-binding protein isoform a
acidic fibroblast growth factor intracellular-binding protein isoform b
acidic fibroblast growth factor intracellular-binding protein isoform X1
activity-regulated cytoskeleton-associated protein
acyl-CoA synthetase family member 3, mitochondrial isoform X3
adhesive plaque matrix protein-like
alpha-2,8-sialyltransferase 8F isoform X2
alpha-2-macroglobulin receptor-associated protein precursor
alpha-L-iduronidase isoform a precursor
alpha-L-iduronidase isoform X1
alpha-L-iduronidase isoform X2
alpha-L-iduronidase isoform X3
alpha-mannosidase 2C1 isoform 1
alpha-mannosidase 2C1 isoform 2
alpha-mannosidase 2C1 isoform 3
alpha-mannosidase 2C1 isoform 4
alpha-mannosidase 2C1 isoform X1
alpha-mannosidase 2C1 isoform X2
alpha-mannosidase 2C1 isoform X3
alpha-mannosidase 2C1 isoform X4
amelogenin, X isoform isoform 1 precursor
amelogenin, X isoform isoform 2
amelogenin, X isoform isoform 3
amelogenin, X isoform isoform X1
amelogenin, Y isoform isoform 1 precursor
amelogenin, Y isoform isoform X1
angiopoietin-like protein 8 precursor
ankyrin repeat domain-containing protein 20B-like
ankyrin repeat domain-containing protein 65 isoform 2
ankyrin repeat domain-containing protein 65 isoform X2
ankyrin repeat domain-containing protein 9
ankyrin repeat domain-containing protein 9
ankyrin repeat domain-containing protein 9

ankyrin repeat domain-containing protein 9 isoform X1
annexin-2 receptor
anoctamin-7 isoform NGEP-short
AP-4 complex accessory subunit tepsin isoform 2
AP-4 complex accessory subunit tepsin isoform X10
AP-4 complex accessory subunit tepsin isoform X1
AP-4 complex accessory subunit tepsin isoform X2
AP-4 complex accessory subunit tepsin isoform X3
AP-4 complex accessory subunit tepsin isoform X4
AP-4 complex accessory subunit tepsin isoform X5
AP-4 complex accessory subunit tepsin isoform X6
AP-4 complex accessory subunit tepsin isoform X7
AP-4 complex accessory subunit tepsin isoform X8
AP-4 complex accessory subunit tepsin isoform X8
AP-4 complex accessory subunit tepsin isoform X8
AP-4 complex accessory subunit tepsin isoform X9
apical junction component 1 homolog
apoptosis regulatory protein Siva isoform 1
apoptosis regulatory protein Siva isoform X1
arf-GAP with GTPase, ANK repeat and PH domain-containing protein 2
arf-GAP with GTPase, ANK repeat and PH domain-containing protein 2-like
arrestin domain-containing protein 5 isoform X2
arrestin domain-containing protein 5 isoform X2
arrestin domain-containing protein 5 isoform X2
arrestin domain-containing protein 5 isoform X3
arrestin domain-containing protein 5 isoform X4
aspartate-rich protein 1 isoform X2
aspartate-rich protein 1 isoform X5
ataxin-1 Alt-ATXN1
atherin-like
ATP synthase subunit delta, mitochondrial precursor
ATP synthase subunit delta, mitochondrial precursor
B9 domain-containing protein 1 isoform a
basic proline-rich protein
basic proline-rich protein-like
basic proline-rich protein-like
basic proline-rich protein-like
basic proline-rich protein-like
basic salivary proline-rich protein 1 isoform 1 preproprotein
basic salivary proline-rich protein 1 isoform 2 precursor
basic salivary proline-rich protein 1 isoform 3 precursor
basic salivary proline-rich protein 2 preproprotein
basic salivary proline-rich protein 3-like isoform X2
basic salivary proline-rich protein 3-like isoform X4
basic salivary proline-rich protein 3 precursor

basic salivary proline-rich protein 4 isoform 1 preproprotein
basic salivary proline-rich protein 4 isoform 2 precursor
basic salivary proline-rich protein 4-like isoform X3
B-cell antigen receptor complex-associated protein alpha chain isoform 2 precursor
bcl-2-binding component 3 isoform 1
beckwith-Wiedemann syndrome chromosomal region 1 candidate gene B protein isoform a
beckwith-Wiedemann syndrome chromosomal region 1 candidate gene B protein isoform b
beckwith-Wiedemann syndrome chromosomal region 1 candidate gene B protein isoform X1
beckwith-Wiedemann syndrome chromosomal region 1 candidate gene B protein isoform X2
beckwith-Wiedemann syndrome chromosomal region 1 candidate gene B protein isoform X3
beckwith-Wiedemann syndrome chromosomal region 1 candidate gene B protein isoform X3
beckwith-Wiedemann syndrome chromosomal region 1 candidate gene B protein isoform X3
beta-lactoglobulin
bone marrow stromal antigen 2 precursor
BRCA1-associated ATM activator 1 isoform X7
BRCA1-associated ATM activator 1 isoform X8
BRISC and BRCA1-A complex member 1 isoform 1
BRISC and BRCA1-A complex member 1 isoform 1
BRISC and BRCA1-A complex member 1 isoform 1
BRISC and BRCA1-A complex member 1 isoform 2
BTB/POZ domain-containing protein KCTD15 isoform X2
cactin
cactin
cactin isoform X1
cactin isoform X2
cadherin-like protein 26 isoform b
cancer/testis antigen 2 isoform LAGE-1b
carnosine synthase 1 isoform 1
carnosine synthase 1 isoform 2
carnosine synthase 1 isoform X1
carnosine synthase 1 isoform X2
carnosine synthase 1 isoform X3
casein kinase II subunit alpha'-interacting protein
caspase recruitment domain-containing protein 8 isoform a
caspase recruitment domain-containing protein 8 isoform a
caspase recruitment domain-containing protein 8 isoform b
caspase recruitment domain-containing protein 8 isoform b
caspase recruitment domain-containing protein 8 isoform b
caspase recruitment domain-containing protein 8 isoform c
caspase recruitment domain-containing protein 8 isoform c
caspase recruitment domain-containing protein 8 isoform e
caspase recruitment domain-containing protein 8 isoform f
caspase recruitment domain-containing protein 8 isoform g
caspase recruitment domain-containing protein 8 isoform h
caspase recruitment domain-containing protein 8 isoform h

caspase recruitment domain-containing protein 8 isoform i
caspase recruitment domain-containing protein 8 isoform X10
caspase recruitment domain-containing protein 8 isoform X10
caspase recruitment domain-containing protein 8 isoform X11
caspase recruitment domain-containing protein 8 isoform X12
caspase recruitment domain-containing protein 8 isoform X12
caspase recruitment domain-containing protein 8 isoform X13
caspase recruitment domain-containing protein 8 isoform X13
caspase recruitment domain-containing protein 8 isoform X14
caspase recruitment domain-containing protein 8 isoform X14
caspase recruitment domain-containing protein 8 isoform X15
caspase recruitment domain-containing protein 8 isoform X16
caspase recruitment domain-containing protein 8 isoform X17
caspase recruitment domain-containing protein 8 isoform X17
caspase recruitment domain-containing protein 8 isoform X18
caspase recruitment domain-containing protein 8 isoform X1
caspase recruitment domain-containing protein 8 isoform X1
caspase recruitment domain-containing protein 8 isoform X1
caspase recruitment domain-containing protein 8 isoform X1
caspase recruitment domain-containing protein 8 isoform X1
caspase recruitment domain-containing protein 8 isoform X1
caspase recruitment domain-containing protein 8 isoform X2
caspase recruitment domain-containing protein 8 isoform X2
caspase recruitment domain-containing protein 8 isoform X3
caspase recruitment domain-containing protein 8 isoform X3
caspase recruitment domain-containing protein 8 isoform X3
caspase recruitment domain-containing protein 8 isoform X4
caspase recruitment domain-containing protein 8 isoform X5
caspase recruitment domain-containing protein 8 isoform X5
caspase recruitment domain-containing protein 8 isoform X6
caspase recruitment domain-containing protein 8 isoform X7
caspase recruitment domain-containing protein 8 isoform X8
caspase recruitment domain-containing protein 8 isoform X8
caspase recruitment domain-containing protein 8 isoform X9
CASP-like protein 4A1 isoform X1
CD160 antigen isoform X1
CD160 antigen isoform X1
CD160 antigen precursor
cell cycle exit and neuronal differentiation protein 1
cementoblastoma-derived protein 1
centrosomal protein of 131 kDa isoform a
centrosomal protein of 131 kDa isoform c
centrosomal protein of 131 kDa isoform d
centrosomal protein of 131 kDa isoform X1
centrosomal protein of 131 kDa isoform X1

centrosomal protein of 131 kDa isoform X2
centrosomal protein of 131 kDa isoform X3
centrosomal protein of 131 kDa isoform X4
centrosomal protein of 131 kDa isoform X5
centrosomal protein of 131 kDa isoform X6
centrosomal protein of 131 kDa isoform X7
charged multivesicular body protein 1a isoform 1
chromatin assembly factor 1 subunit A
chromatin assembly factor 1 subunit A isoform X1
chromatin assembly factor 1 subunit A isoform X2
chromatin assembly factor 1 subunit A isoform X3
chromosome transmission fidelity protein 18 homolog isoform X6
cilia- and flagella-associated protein 74
cilia- and flagella-associated protein 74 isoform X1
cilia- and flagella-associated protein 74 isoform X2
cilia- and flagella-associated protein 74 isoform X3
cilia- and flagella-associated protein 74 isoform X5
cilia- and flagella-associated protein 74 isoform X5
cilia- and flagella-associated protein 74 isoform X6
claudin domain-containing protein 2 isoform X1
claudin domain-containing protein 2 isoform X1
claudin domain-containing protein 2 isoform X2
claudin domain-containing protein 2 isoform X3
coatomer subunit epsilon isoform a
coatomer subunit epsilon isoform b
coatomer subunit epsilon isoform c
coatomer subunit epsilon isoform d
coatomer subunit epsilon isoform X1
cob(I)yrinic acid a,c-diamide adenosyltransferase, mitochondrial isoform X2
coiled-coil domain-containing protein 124
coiled-coil domain-containing protein 124
coiled-coil domain-containing protein 130 isoform a
coiled-coil domain-containing protein 130 isoform a
coiled-coil domain-containing protein 130 isoform a
coiled-coil domain-containing protein 130 isoform a
coiled-coil domain-containing protein 130 isoform b
coiled-coil domain-containing protein 130 isoform b
coiled-coil domain-containing protein 130 isoform b
coiled-coil domain-containing protein 130 isoform c
coiled-coil domain-containing protein 130 isoform X1
coiled-coil domain-containing protein 130 isoform X2
coiled-coil domain-containing protein 137
coiled-coil domain-containing protein 137 isoform X1
coiled-coil domain-containing protein 137 isoform X2
coiled-coil domain-containing protein 140

coiled-coil domain-containing protein 144B-like
coiled-coil domain-containing protein 151 isoform 1
coiled-coil domain-containing protein 151 isoform 2
coiled-coil domain-containing protein 151 isoform 3
coiled-coil domain-containing protein 151 isoform X1
coiled-coil domain-containing protein 183
coiled-coil domain-containing protein 187
coiled-coil domain-containing protein 187 isoform X1
coiled-coil domain-containing protein 187 isoform X2
coiled-coil domain-containing protein 187 isoform X3
coiled-coil domain-containing protein 187 isoform X4
coiled-coil domain-containing protein 194
coiled-coil domain-containing protein 27
coiled-coil domain-containing protein 27 isoform X1
coiled-coil domain-containing protein 27 isoform X2
coiled-coil domain-containing protein 27 isoform X3
coiled-coil domain-containing protein 27 isoform X4
coiled-coil domain-containing protein 27 isoform X5
coiled-coil domain-containing protein 27 isoform X6
coiled-coil domain-containing protein 78
coiled-coil domain-containing protein 78 isoform X10
coiled-coil domain-containing protein 78 isoform X11
coiled-coil domain-containing protein 78 isoform X12
coiled-coil domain-containing protein 78 isoform X13
coiled-coil domain-containing protein 78 isoform X14
coiled-coil domain-containing protein 78 isoform X15
coiled-coil domain-containing protein 78 isoform X16
coiled-coil domain-containing protein 78 isoform X17
coiled-coil domain-containing protein 78 isoform X18
coiled-coil domain-containing protein 78 isoform X19
coiled-coil domain-containing protein 78 isoform X1
coiled-coil domain-containing protein 78 isoform X20
coiled-coil domain-containing protein 78 isoform X21
coiled-coil domain-containing protein 78 isoform X22
coiled-coil domain-containing protein 78 isoform X2
coiled-coil domain-containing protein 78 isoform X3
coiled-coil domain-containing protein 78 isoform X4
coiled-coil domain-containing protein 78 isoform X5
coiled-coil domain-containing protein 78 isoform X6
coiled-coil domain-containing protein 78 isoform X7
coiled-coil domain-containing protein 78 isoform X8
coiled-coil domain-containing protein 78 isoform X9
coiled-coil domain-containing protein 86-like
collagen alpha-1(I) chain
collagen alpha-1(I) chain-like

collagen alpha-1(III) chain-like
collagen alpha-1(III) chain-like
collagen alpha-1(III) chain-like
collagen alpha-1(VII) chain-like isoform X1
collagen alpha-2(I) chain-like
collagen alpha-2(IV) chain-like
collagen alpha-3(IX) chain-like
collagen alpha-3(IX) chain-like
collagen alpha-3(IX) chain-like
collagen alpha-3(IX) chain-like
collagen alpha-3(IX) chain-like
collagen alpha-3(IX) chain-like
COMM domain-containing protein 4 isoform X1
COMM domain-containing protein 4 isoform X6
COMM domain-containing protein 4-like isoform X1
complement component 1 Q subcomponent-binding protein, mitochondrial precursor
cryptic family protein 1B isoform X1
cyclic AMP-responsive element-binding protein 5 isoform X7
cysteine and histidine-rich protein 1 isoform 2 precursor
cysteine and histidine-rich protein 1 isoform 2 precursor
cysteine-rich PAK1 inhibitor
cysteine-rich tail protein 1
cytochrome b-245 light chain
cytochrome b-245 light chain isoform X1
cytoplasmic tRNA 2-thiolation protein 2 isoform 1
cytoplasmic tRNA 2-thiolation protein 2 isoform 2
cytoplasmic tRNA 2-thiolation protein 2 isoform 3
cytoplasmic tRNA 2-thiolation protein 2 isoform 4
cytoplasmic tRNA 2-thiolation protein 2 isoform X1
D-amino acid oxidase activator isoform 1
DAZ-associated protein 1 isoform e
DAZ-associated protein 1 isoform X8
DAZ-associated protein 1 isoform X9
D-beta-hydroxybutyrate dehydrogenase, mitochondrial isoform X5
DDB1- and CUL4-associated factor 15
death-associated protein 1 isoform 1
delta-like protein 3
dendritic cell nuclear protein 1
deoxyhypusine hydroxylase
deoxyhypusine hydroxylase
deoxyhypusine hydroxylase isoform X1
dexamethasone-induced protein isoform X1
D-glutamate cyclase, mitochondrial isoform a precursor
D-glutamate cyclase, mitochondrial isoform a precursor
D-glutamate cyclase, mitochondrial isoform a precursor

D-glutamate cyclase, mitochondrial isoform a precursor
D-glutamate cyclase, mitochondrial isoform a precursor
D-glutamate cyclase, mitochondrial isoform b precursor
D-glutamate cyclase, mitochondrial isoform b precursor
D-glutamate cyclase, mitochondrial isoform b precursor
D-glutamate cyclase, mitochondrial isoform b precursor
D-glutamate cyclase, mitochondrial isoform c
D-glutamate cyclase, mitochondrial isoform d
D-glutamate cyclase, mitochondrial isoform e
diphosphomevalonate decarboxylase
diphosphomevalonate decarboxylase isoform X1
diphosphomevalonate decarboxylase isoform X2
diphosphomevalonate decarboxylase isoform X3
diphosphomevalonate decarboxylase isoform X4
DNA-directed RNA polymerase II subunit 1-like
DNA-directed RNA polymerase II subunit RPB1-like
DNA-directed RNA polymerases I, II, and III subunit RPABC1 isoform a
DNA-directed RNA polymerases I, II, and III subunit RPABC1 isoform X1
DNL-type zinc finger protein
dual specificity protein phosphatase 22 isoform X1
dynein assembly factor 3, axonemal isoform 1
dynein assembly factor 3, axonemal isoform 2
dynein assembly factor 3, axonemal isoform 3
dynein assembly factor 3, axonemal isoform 4
dynein assembly factor 5, axonemal
dynein assembly factor 5, axonemal isoform X1
dynein assembly factor 5, axonemal isoform X2
E3 ubiquitin-protein ligase MARCH2 isoform X4
E3 ubiquitin-protein ligase MARCH3 isoform X4
E3 ubiquitin-protein ligase TM129 isoform b
E3 ubiquitin-protein ligase TM129 isoform X1
E3 ubiquitin-protein ligase TM129 isoform X1
E3 ubiquitin-protein ligase TM129 isoform X1
E3 ubiquitin-protein ligase TM129 isoform X1
E3 ubiquitin-protein ligase ZNF598
E3 ubiquitin-protein transferase MAEA isoform 4
elongation of very long chain fatty acids protein 7 isoform X3
elongation of very long chain fatty acids protein 7 isoform X4
endogenous Bornavirus-like nucleoprotein 1
endogenous Bornavirus-like nucleoprotein 2
endogenous retroviral envelope protein HEMO precursor
endogenous retrovirus group 3 member 1 Env polyprotein precursor
endogenous retrovirus group K member 19 Env polyprotein-like
endogenous retrovirus group K member 8 Gag polyprotein-like
endonuclease 8-like 1 isoform X3

endonuclease 8-like 1 isoform X5
endonuclease III-like protein 1 isoform 1
endonuclease III-like protein 1 isoform 2
endonuclease III-like protein 1 isoform 3
endonuclease III-like protein 1 isoform X1
endonuclease V isoform X20
endonuclease V isoform X20
endonuclease V isoform X20
endonuclease V isoform X20
endosomal/lysosomal potassium channel TMEM175 isoform 1
endosomal/lysosomal potassium channel TMEM175 isoform 2
endosomal/lysosomal potassium channel TMEM175 isoform 2
endosomal/lysosomal potassium channel TMEM175 isoform 2
endosomal/lysosomal potassium channel TMEM175 isoform 3
endosomal/lysosomal potassium channel TMEM175 isoform 3
endosomal/lysosomal potassium channel TMEM175 isoform 3
endosomal/lysosomal potassium channel TMEM175 isoform X1
endosomal/lysosomal potassium channel TMEM175 isoform X2
endosomal/lysosomal potassium channel TMEM175 isoform X3
endosomal/lysosomal potassium channel TMEM175 isoform X3
endosomal/lysosomal potassium channel TMEM175 isoform X3
endosomal/lysosomal potassium channel TMEM175 isoform X3
endosomal/lysosomal potassium channel TMEM175 isoform X4
endosomal/lysosomal potassium channel TMEM175 isoform X5
endosomal/lysosomal potassium channel TMEM175 isoform X5
endosomal/lysosomal potassium channel TMEM175 isoform X5
endosomal/lysosomal potassium channel TMEM175 isoform X6
endosomal/lysosomal potassium channel TMEM175 isoform X7
enolase-phosphatase E1
envoplakin-like protein isoform X4
epididymal-specific lipocalin-10 precursor
epididymal-specific lipocalin-6 precursor
epididymal-specific lipocalin-8 isoform 1 precursor
epididymal-specific lipocalin-8 isoform 2
epididymal-specific lipocalin-8 isoform X1
ER membrane protein complex subunit 10 isoform 1 precursor
ER membrane protein complex subunit 10 isoform 2 precursor
ER membrane protein complex subunit 10 isoform X1
ER membrane protein complex subunit 10 isoform X2
ES1 protein homolog, mitochondrial isoform X1
ES1 protein homolog, mitochondrial isoform X1
ES1 protein homolog, mitochondrial isoform X2
ES1 protein homolog, mitochondrial isoform X2
ES1 protein homolog, mitochondrial isoform X3
ES1 protein homolog, mitochondrial isoform X4

ES1 protein homolog, mitochondrial isoform X6
ES1 protein homolog, mitochondrial isoform X7
ES1 protein homolog, mitochondrial isoform X7
ES1 protein homolog, mitochondrial isoform X8
exonuclease mut-7 homolog isoform b
FAD-linked sulfhydryl oxidase ALR
FAM231A/C-like protein LOC102723383
FAM231A/C-like protein LOC102723383
Fanconi anemia core complex-associated protein 20 isoform 1
Fanconi anemia core complex-associated protein 20 isoform 2
Fanconi anemia core complex-associated protein 20 isoform 3
Fanconi anemia core complex-associated protein 20 isoform 4
Fanconi anemia core complex-associated protein 20 isoform 6
Fanconi anemia core complex-associated protein 20 isoform 8
Fanconi anemia core complex-associated protein 20 isoform X1
Fanconi anemia core complex-associated protein 20 isoform X2
Fanconi anemia core complex-associated protein 20 isoform X3
Fanconi anemia core complex-associated protein 20 isoform X4
Fanconi anemia core complex-associated protein 20 isoform X5
Fanconi anemia core complex-associated protein 20 isoform X6
Fanconi anemia core complex-associated protein 20 isoform X7
fetal and adult testis-expressed transcript protein
fibronectin type III domain-containing protein 10 precursor
fibronectin type III domain-containing protein 11
fibronectin type III domain-containing protein 11
fibronectin type III domain-containing protein 11
fibronectin type III domain-containing protein 11 isoform X1
fibronectin type III domain-containing protein 11 isoform X2
flocculation protein FLO11-like
formiminotransferase N-terminal subdomain-containing protein isoform X1
formiminotransferase N-terminal subdomain-containing protein isoform X1
formiminotransferase N-terminal subdomain-containing protein isoform X1
formin-2-like
formin-2-like
formin-2-like
FOXL2 neighbor protein
FOXL2 neighbor protein isoform X1
FOXL2 neighbor protein isoform X1
FOXL2 neighbor protein isoform X2
gamma-interferon-inducible lysosomal thiol reductase preproprotein
glucose-dependent insulinotropic receptor-like
glutamate-rich protein 1 isoform X21
glutamate-rich protein 1 isoform X21
glutamate-rich protein 1 isoform X21
glutamate-rich protein 1 isoform X21

glutamate-rich protein 1 isoform X21
glutamate-rich protein 2 isoform 3
glutamine amidotransferase-like class 1 domain-containing protein 1 isoform 1 precursor
glutamine amidotransferase-like class 1 domain-containing protein 1 isoform 2
glutamine amidotransferase-like class 1 domain-containing protein 1 isoform 3
glutamine amidotransferase-like class 1 domain-containing protein 1 isoform 4 precursor
glutamine amidotransferase-like class 1 domain-containing protein 1 isoform 6
glutamine amidotransferase-like class 1 domain-containing protein 1 isoform 7
glutamine amidotransferase-like class 1 domain-containing protein 1 isoform X1
glutamine amidotransferase-like class 1 domain-containing protein 1 isoform X2
glutamine amidotransferase-like class 1 domain-containing protein 1 isoform X3
glutamine amidotransferase-like class 1 domain-containing protein 1 isoform X4
glutamine amidotransferase-like class 1 domain-containing protein 1 isoform X5
glutamine amidotransferase-like class 1 domain-containing protein 1 isoform X5
glutamine amidotransferase-like class 1 domain-containing protein 1 isoform X6
glutamine amidotransferase-like class 1 domain-containing protein 1 isoform X7
glutamine amidotransferase-like class 1 domain-containing protein 3A, mitochondrial isoform 1 precursor
glutamine amidotransferase-like class 1 domain-containing protein 3A, mitochondrial isoform 2 precursor
glutamine amidotransferase-like class 1 domain-containing protein 3A, mitochondrial isoform 3 precursor
glutamine amidotransferase-like class 1 domain-containing protein 3A, mitochondrial isoform 4 precursor
glutamine-dependent NAD(+) synthetase
glycodelin isoform 1 precursor
glycodelin isoform 1 precursor
glycodelin isoform 2 precursor
glycodelin isoform X1
glycodelin isoform X1
glycodelin isoform X1
glycodelin isoform X2
glycodelin isoform X3
glycodelin isoform X4
glycoprotein hormone alpha-2 isoform X1
glycoprotein Xg isoform 1 precursor
glycoprotein Xg isoform 2 precursor
glycoprotein Xg isoform 3 precursor
glycoprotein Xg isoform X1
glycoprotein Xg isoform X2
glycosylphosphatidylinositol-anchored high density lipoprotein-binding protein 1 isoform 1 precursor
golgin subfamily A member 6-like protein 9 isoform 2
Golgi to ER traffic protein 4 homolog
GPI mannosyltransferase 4 isoform X3
GPI mannosyltransferase 4 isoform X3
G-protein coupled receptor 143
G-protein coupled receptor 143 isoform X1
G-protein coupled receptor 143 isoform X2
G-protein coupled receptor 143 isoform X2

grainyhead-like protein 2 homolog isoform X3
granulysin isoform 1 precursor
granulysin isoform X1
granulysin isoform X2
granulysin isoform X3
GSK-3-binding protein FRAT2
guanidinoacetate N-methyltransferase isoform a
guanidinoacetate N-methyltransferase isoform b
H19 opposite tumor suppressor
helicase SRCAP-like
helix-loop-helix protein 2 isoform X1
heme transporter HRG1 isoform X1
heme transporter HRG1 isoform X1
HERV-H LTR-associating protein 3 isoform X1
histidine-rich protein PFHRP-II-like
histone-lysine N-methyltransferase, H3 lysine-79 specific
histone-lysine N-methyltransferase, H3 lysine-79 specific isoform X1
histone-lysine N-methyltransferase, H3 lysine-79 specific isoform X1
histone-lysine N-methyltransferase, H3 lysine-79 specific isoform X2
histone-lysine N-methyltransferase, H3 lysine-79 specific isoform X3
histone-lysine N-methyltransferase, H3 lysine-79 specific isoform X4
histone-lysine N-methyltransferase, H3 lysine-79 specific isoform X5
histone RNA hairpin-binding protein isoform 1
histone RNA hairpin-binding protein isoform 2
histone RNA hairpin-binding protein isoform 3
homeobox protein HMX1 isoform 2
homeobox protein Hox-D13 isoform X2
immunoglobulin superfamily member 23
immunoglobulin superfamily member 23 isoform X1
immunoglobulin superfamily member 23 isoform X2
immunoglobulin superfamily member 23 isoform X3
immunoglobulin superfamily member 23 isoform X4
immunoglobulin superfamily member 23 isoform X4
immunoglobulin superfamily member 23 isoform X4
immunoglobulin superfamily member 23 isoform X5
immunoglobulin superfamily member 23 isoform X6
immunoglobulin superfamily member 23 isoform X7
inositol-3-phosphate synthase 1 isoform 1
inositol-3-phosphate synthase 1 isoform 2
inositol-3-phosphate synthase 1 isoform X1
inositol-3-phosphate synthase 1 isoform X2
inositol-3-phosphate synthase 1 isoform X3
inositol-3-phosphate synthase 1 isoform X4
integrator complex subunit 1 isoform X4
interleukin-11 isoform 1 precursor

interleukin-12 receptor subunit beta-1 isoform 1 precursor
interleukin-12 receptor subunit beta-1 isoform 2 precursor
interleukin-12 receptor subunit beta-1 isoform 3 precursor
interleukin-12 receptor subunit beta-1 isoform 4
interleukin-12 receptor subunit beta-1 isoform X10
interleukin-12 receptor subunit beta-1 isoform X11
interleukin-12 receptor subunit beta-1 isoform X12
interleukin-12 receptor subunit beta-1 isoform X13
interleukin-12 receptor subunit beta-1 isoform X14
interleukin-12 receptor subunit beta-1 isoform X1
interleukin-12 receptor subunit beta-1 isoform X2
interleukin-12 receptor subunit beta-1 isoform X3
interleukin-12 receptor subunit beta-1 isoform X4
interleukin-12 receptor subunit beta-1 isoform X5
interleukin-12 receptor subunit beta-1 isoform X6
interleukin-12 receptor subunit beta-1 isoform X7
interleukin-12 receptor subunit beta-1 isoform X8
interleukin-12 receptor subunit beta-1 isoform X9
interleukin-24 isoform 4 precursor
interleukin-31 precursor
interleukin-3 precursor
interleukin-9 receptor isoform X15
interleukin-9 receptor isoform X15
isthmin-2 isoform 3 precursor
izumo sperm-egg fusion protein 4 isoform 1 precursor
izumo sperm-egg fusion protein 4 isoform 3 precursor
izumo sperm-egg fusion protein 4 isoform X1
izumo sperm-egg fusion protein 4 isoform X2
izumo sperm-egg fusion protein 4 isoform X3
izumo sperm-egg fusion protein 4 isoform X4
jmjC domain-containing protein 8 isoform 2 precursor
jmjC domain-containing protein 8 isoform 3 precursor
jmjC domain-containing protein 8 isoform 5 precursor
junctional sarcoplasmic reticulum protein 1
junctional sarcoplasmic reticulum protein 1 isoform X1
junctional sarcoplasmic reticulum protein 1 isoform X2
junctional sarcoplasmic reticulum protein 1 isoform X3
keratin-associated protein 5-1
kxDL motif-containing protein 1
kxDL motif-containing protein 1
kxDL motif-containing protein 1
kxDL motif-containing protein 1 isoform X1
kxDL motif-containing protein 1 isoform X1
kxDL motif-containing protein 1 isoform X1
kxDL motif-containing protein 1 isoform X1

laforin-like
lamin tail domain-containing protein 2 isoform X4
lck-interacting transmembrane adapter 1 isoform 1 precursor
lck-interacting transmembrane adapter 1 isoform 2 precursor
lck-interacting transmembrane adapter 1 isoform 2 precursor
leucine repeat adapter protein 25 isoform 1
leucine repeat adapter protein 25 isoform 2
leucine repeat adapter protein 25 isoform 3
leucine-rich colipase-like protein 1 isoform X1
leucine-rich colipase-like protein 1 isoform X2
leucine-rich colipase-like protein 1 isoform X3
leucine-rich colipase-like protein 1 isoform X4
leucine-rich colipase-like protein 1 isoform X4
leucine-rich colipase-like protein 1 isoform X4
leucine-rich colipase-like protein 1 isoform X4
leucine-rich colipase-like protein 1 precursor
leucine-rich repeat-containing protein 25 isoform X1
leucine-rich repeat-containing protein 25 precursor
leucine-rich repeat-containing protein 53 isoform X3
leukocyte receptor cluster member 9 isoform 1
leukocyte receptor cluster member 9 isoform 2
leukocyte surface antigen CD47 isoform X3
linker for activation of T-cells family member 2 isoform X1
linker for activation of T-cells family member 2 precursor
linker for activation of T-cells family member 2 precursor
linker for activation of T-cells family member 2 precursor
lipocalin-like 1 protein
lon protease homolog, mitochondrial isoform X1
loricrin
ly6/PLAUR domain-containing protein 8 preproprotein
lymphocyte antigen 6H isoform b precursor
lymphocyte antigen 6H isoform b precursor
lymphocyte antigen 6H isoform X1
lymphocyte function-associated antigen 3 isoform 1
lymphocyte function-associated antigen 3 isoform 2
lymphocyte function-associated antigen 3 isoform X1
MAGE-like protein 2
MAGE-like protein 2 isoform X2
MAGE-like protein 2 isoform X2
MAGE-like protein 2 isoform X3
MAGE-like protein 2 isoform X4
MAPK-interacting and spindle-stabilizing protein-like
matrix remodeling-associated protein 8 isoform 1 precursor
matrix remodeling-associated protein 8 isoform 2 precursor
matrix remodeling-associated protein 8 isoform 2 precursor

matrix remodeling-associated protein 8 isoform 3 precursor
matrix remodeling-associated protein 8 isoform 4
matrix remodeling-associated protein 8 isoform X1
matrix remodeling-associated protein 8 isoform X2
mediator of RNA polymerase II transcription subunit 16
mediator of RNA polymerase II transcription subunit 16 isoform X1
mediator of RNA polymerase II transcription subunit 16 isoform X2
mediator of RNA polymerase II transcription subunit 22 isoform b
melanoma-associated antigen C1-like
melanoma-associated antigen C1-like
melanoma-associated antigen C1-like
melanoma-associated antigen C1-like
membralin isoform 1
membralin isoform 2
membralin isoform X1
membralin isoform X2
membralin isoform X3
membralin isoform X4
membralin isoform X5
membralin isoform X6
membrane progestin receptor delta isoform 4
membrane progestin receptor delta isoform 7
membrane progestin receptor delta isoform 7
membrane progestin receptor delta isoform 7
membrane progestin receptor delta isoform 7
membrane progestin receptor delta isoform 7
membrane progestin receptor delta isoform X5
membrane progestin receptor delta isoform X5
membrane progestin receptor delta isoform X5
membrane progestin receptor delta isoform X5
mesothelin isoform 1 preproprotein
mesothelin isoform 2 preproprotein
methyl-CpG-binding domain protein 6-like, partial
methyltransferase-like 26 isoform a
methyltransferase-like 26 isoform b
methyltransferase-like 26 isoform g
methyltransferase-like 26 isoform h
methyltransferase-like 26 isoform X1
methyltransferase-like 26 isoform X2
mitochondrial basic amino acids transporter isoform X5
mitochondrial import inner membrane translocase subunit Tim29
mitogen-activated protein kinase 7-like
mitotic interactor and substrate of PLK1
mitotic interactor and substrate of PLK1 isoform X1
mitotic interactor and substrate of PLK1 isoform X1

mixed lineage kinase domain-like protein isoform 2
mixed lineage kinase domain-like protein isoform X2
MRG/MORF4L-binding protein
mucin-1 isoform 8 precursor
mucin-1-like
mucin-1-like
mucin-1-like
mucin-5AC-like isoform X2
mucin-7 precursor
mucin-7 precursor
mucin-7 precursor
myeloid-derived growth factor isoform X1
myeloid-derived growth factor precursor
myeloma-overexpressed gene protein isoform 1
myeloma-overexpressed gene protein isoform 1
myeloma-overexpressed gene protein isoform 2
myeloma-overexpressed gene protein isoform 2
myeloma-overexpressed gene protein isoform 2
myeloma-overexpressed gene protein isoform 2
myosin heavy chain IB-like
N-acetylglucosamine-1-phosphotransferase subunit gamma isoform X1
N-acetylglucosamine-1-phosphotransferase subunit gamma isoform X2
N-acetylglucosamine-1-phosphotransferase subunit gamma precursor
N-acetyltransferase 14
NAD-dependent protein deacetylase sirtuin-6 isoform 4
NAD-dependent protein deacetylase sirtuin-6 isoform 7
NAD-dependent protein deacetylase sirtuin-6 isoform 8
NADH dehydrogenase [ubiquinone] 1 beta subcomplex subunit 10
NADH dehydrogenase [ubiquinone] flavoprotein 1, mitochondrial isoform 1 precursor
NADH dehydrogenase [ubiquinone] flavoprotein 1, mitochondrial isoform 2 precursor
NADH dehydrogenase [ubiquinone] iron-sulfur protein 7, mitochondrial isoform 1
NADH dehydrogenase [ubiquinone] iron-sulfur protein 7, mitochondrial isoform X1
NADH dehydrogenase [ubiquinone] iron-sulfur protein 7, mitochondrial isoform X2
NADH dehydrogenase [ubiquinone] iron-sulfur protein 7, mitochondrial isoform X3
NADH dehydrogenase [ubiquinone] iron-sulfur protein 8, mitochondrial precursor
nascent polypeptide-associated complex subunit alpha, muscle-specific form-like
nascent polypeptide-associated complex subunit alpha, muscle-specific form-like
natural cytotoxicity triggering receptor 3 ligand 1-like isoform X1
N-cym protein isoform X1
negative elongation factor B
netrin-G2 isoform X7
neural proliferation differentiation and control protein 1 precursor
neurocalcin-delta isoform X1
neuropeptide-like protein C4orf48 isoform X1
neuropeptide W preproprotein

nicalin isoform 1 precursor
nicalin isoform 2 precursor
non-histone chromosomal protein HMG-14 isoform X1
non-histone chromosomal protein HMG-14 isoform X1
non-histone chromosomal protein HMG-14 isoform X1
non-histone chromosomal protein HMG-14 isoform X1
non-histone chromosomal protein HMG-14 isoform X1
non-histone chromosomal protein HMG-14 isoform X1
non-histone chromosomal protein HMG-14 isoform X1
non-histone chromosomal protein HMG-14 isoform X1
non-histone chromosomal protein HMG-14 isoform X1
non-histone chromosomal protein HMG-14 isoform X1
non-histone chromosomal protein HMG-14 isoform X1
non-histone chromosomal protein HMG-14 isoform X1
nuclear pore complex-interacting protein family member A1
nuclear pore complex-interacting protein family member A1-like isoform X3
nuclear pore complex-interacting protein family member A1-like isoform X4
nuclear pore complex-interacting protein family member A1-like isoform X5
nuclear pore complex-interacting protein family member A1-like isoform X7
nuclear pore complex-interacting protein family member A2
nuclear pore complex-interacting protein family member A2 isoform X10
nuclear pore complex-interacting protein family member A2 isoform X2
nuclear pore complex-interacting protein family member A2 isoform X5
nuclear pore complex-interacting protein family member A2 isoform X9
nuclear pore complex-interacting protein family member A3
nuclear pore complex-interacting protein family member A3 isoform X2
nuclear pore complex-interacting protein family member A3 isoform X4
nuclear pore complex-interacting protein family member A3 isoform X8
nuclear pore complex-interacting protein family member A3 isoform X9
nuclear pore complex-interacting protein family member A5 isoform 1
nuclear pore complex-interacting protein family member A5 isoform X4
nuclear pore complex-interacting protein family member A5-like isoform X8
nuclear pore complex-interacting protein family member A7 isoform X1
nuclear pore complex-interacting protein family member A7 isoform X2
nuclear pore complex-interacting protein family member A7 isoform X3
nuclear pore complex-interacting protein family member A7 isoform X4
nuclear pore complex-interacting protein family member A7 isoform X4
nuclear pore complex-interacting protein family member A7 isoform X5
nuclear pore complex-interacting protein family member A7 isoform X6
nuclear pore complex-interacting protein family member A7 isoform X6
nuclear pore complex-interacting protein family member A7 isoform X7
nuclear pore complex-interacting protein family member A7 isoform X8
nuclear pore complex-interacting protein family member A7 isoform X9
nuclear pore complex-interacting protein family member A7-like isoform X1
nuclear pore complex-interacting protein family member A7-like isoform X2

nuclear pore complex-interacting protein family member A7-like isoform X6
nuclear pore complex-interacting protein family member A8
nuclear pore complex-interacting protein family member A8
nuclear pore complex-interacting protein family member B11 isoform X3
nuclear pore complex-interacting protein family member B15 isoform X1
nuclear pore complex-interacting protein family member B15 isoform X2
nuclear pore complex-interacting protein family member B15 isoform X2
nuclear pore complex-interacting protein family member B15 isoform X3
nuclear pore complex-interacting protein family member B15 isoform X4
nuclear pore complex-interacting protein family member B15 precursor
nuclear pore complex-interacting protein family member B4 isoform X3
nuclear pore complex-interacting protein family member B4 isoform X6
nuclear pore complex-interacting protein family member B4 isoform X7
nuclear pore complex-interacting protein family member B4 isoform X7
nuclear pore complex-interacting protein family member B6 isoform X2
nuclear pore complex-interacting protein family member B6 isoform X3
nuclear pore complex-interacting protein family member B6 isoform X3
nuclear pore complex-interacting protein family member B6 isoform X3
nuclear pore complex-interacting protein family member B6 isoform X4
nuclear pore complex-interacting protein family member B6 isoform X5
nuclear pore complex-interacting protein family member B6 isoform X7
nuclear pore complex-interacting protein family member B8 isoform X4
nuclear pore complex-interacting protein family member B8 isoform X5
nuclear pore complex-interacting protein family member B8 isoform X5
nuclear pore complex-interacting protein family member B8 isoform X6
nuclear pore complex-interacting protein family member B8 isoform X7
nuclear pore complex-interacting protein family member B9 isoform 2
nuclear pore complex-interacting protein family member B9 isoform X11
nuclear pore complex-interacting protein family member B9 isoform X12
nuclear pore complex-interacting protein family member B9 isoform X13
nuclear pore complex-interacting protein family member B9 isoform X14
nuclear pore complex-interacting protein family member B9 isoform X5
nuclear pore complex-interacting protein family member B9 isoform X6
nuclear pore complex-interacting protein family member B9 isoform X7
nuclear pore complex-interacting protein family member B9 isoform X7
nuclear pore complex-interacting protein family member B9 isoform X7
nuclear pore complex-interacting protein family member B9 isoform X9
nuclear receptor subfamily 4 group A member 1 isoform X4
nuclear receptor subfamily 4 group A member 1 isoform X5
nucleoside diphosphate-linked moiety X motif 17
nucleoside diphosphate-linked moiety X motif 17 isoform X1
nucleoside diphosphate-linked moiety X motif 17 isoform X2
nucleoside diphosphate-linked moiety X motif 17 isoform X3
nutritionally-regulated adipose and cardiac enriched protein homolog isoform a
nutritionally-regulated adipose and cardiac enriched protein homolog isoform b

nutritionally-regulated adipose and cardiac enriched protein homolog isoform b
nutritionally-regulated adipose and cardiac enriched protein homolog isoform X1
nutritionally-regulated adipose and cardiac enriched protein homolog isoform X1
odontogenesis associated phosphoprotein isoform 1 precursor
odontogenesis associated phosphoprotein isoform X1
opiorphin prepropeptide isoform 1 precursor
palmitoleoyl-protein carboxylesterase NOTUM precursor
paralemmin-3 isoform X2
paraneoplastic antigen Ma6E-like
peroxiredoxin-5, mitochondrial isoform b precursor
peroxiredoxin-5, mitochondrial isoform d precursor
peroxiredoxin-5, mitochondrial isoform L precursor
peroxiredoxin-5, mitochondrial isoform S
peroxisome biogenesis factor 10 isoform X1
phenylalanine--tRNA ligase alpha subunit isoform X1
phosphatidylinositol 4,5-bisphosphate 5-phosphatase A-like
phospholipase A2 inhibitor and Ly6/PLAUR domain-containing protein-like
phospholipase A2 inhibitor and Ly6/PLAUR domain-containing protein-like
phospholipase A2 inhibitor and Ly6/PLAUR domain-containing protein-like
phospholipase A2 inhibitor and Ly6/PLAUR domain-containing protein-like
phospholipase A2 inhibitor and Ly6/PLAUR domain-containing protein-like
phospholipase A2 inhibitor and Ly6/PLAUR domain-containing protein-like
platelet-activating factor acetylhydrolase IB subunit alpha isoform X4
platelet endothelial aggregation receptor 1-like
porimin precursor
post-GPI attachment to proteins factor 2 isoform 10
post-GPI attachment to proteins factor 2 isoform 13
post-GPI attachment to proteins factor 2 isoform 14
post-GPI attachment to proteins factor 2 isoform X20
post-GPI attachment to proteins factor 2 isoform X20
post-GPI attachment to proteins factor 2 isoform X20
post-GPI attachment to proteins factor 2 isoform X21
pre-mRNA-splicing factor cwc22-like
pre T-cell antigen receptor alpha isoform X3
pre T-cell antigen receptor alpha isoform X4
pre T-cell antigen receptor alpha isoform X5
proapoptotic nucleolar protein 1
probable gluconokinase isoform X5
probable inactive tRNA-specific adenosine deaminase-like protein 3 isoform 1
probable inactive tRNA-specific adenosine deaminase-like protein 3 isoform 2
probable palmitoyltransferase ZDHHC11 isoform X14
probable palmitoyltransferase ZDHHC11 isoform X15
probable serine/threonine-protein kinase DDB_G0277071 isoform X2
probable serine/threonine-protein kinase DDB_G0277071 isoform X3
probable serine/threonine-protein kinase DDB_G0277071 isoform X3

probable serine/threonine-protein kinase DDB_G0277071 isoform X4
proline-rich AKT1 substrate 1 isoform a
proline-rich AKT1 substrate 1 isoform b
proline-rich AKT1 substrate 1 isoform b
proline-rich AKT1 substrate 1 isoform b
proline-rich AKT1 substrate 1 isoform b
proline-rich extensin-like protein EPR1
proline-rich extensin-like protein EPR1
proline-rich protein 18
proline-rich protein 18-like
proline-rich protein 20A
proline-rich protein 20B
proline-rich protein 20C
proline-rich protein 20D
proline-rich protein 20E
proline-rich protein 25
proline-rich protein 26 isoform 1
proline-rich protein 26 isoform 2
proline-rich protein 26 isoform 3
proline-rich protein 26 isoform X1
proline-rich protein 26 isoform X2
proline-rich protein 26 isoform X3
proline-rich protein 31
proline-rich protein 36-like isoform X1
proline-rich protein 36-like isoform X1
proline-rich protein, Y-linked
proline-rich protein, Y-linked isoform X1
proline-rich protein, Y-linked isoform X1
proline-rich proteoglycan 2 isoform X4
proline-rich proteoglycan 2-like
proline-rich proteoglycan 2-like
proteasomal ubiquitin receptor ADRM1 isoform 1
proteasomal ubiquitin receptor ADRM1 isoform 1
proteasomal ubiquitin receptor ADRM1 isoform 2
proteasomal ubiquitin receptor ADRM1 isoform 2
proteasomal ubiquitin receptor ADRM1 isoform X1
proteasomal ubiquitin receptor ADRM1 isoform X1
proteasomal ubiquitin receptor ADRM1 isoform X2
proteasomal ubiquitin receptor ADRM1 isoform X2
proteasomal ubiquitin receptor ADRM1 isoform X3
proteasomal ubiquitin receptor ADRM1 isoform X4
proteasomal ubiquitin receptor ADRM1 isoform X5
proteasomal ubiquitin receptor ADRM1 isoform X6
proteasomal ubiquitin receptor ADRM1 isoform X7
proteasomal ubiquitin receptor ADRM1 isoform X8

protein ALEX isoform Alex
protein amnionless isoform X1
protein amnionless isoform X2
protein amnionless isoform X2
protein amnionless precursor
protein app1-like
protein app1-like
protein CEI isoform X1
protein CROC-4 isoform 1
protein CROC-4 isoform X1
protein CROC-4 isoform X1
protein CROC-4 isoform X2
protein CROC-4 isoform X3
protein CROC-4 isoform X3
protein CROC-4 isoform X3
protein CROC-4 isoform X3
protein CROC-4 isoform X3
protein crumbs homolog 3 isoform X1
protein Dok-7 isoform 1
protein Dok-7 isoform 2
protein Dok-7 isoform 3
protein Dok-7 isoform 4
protein Dok-7 isoform X1
protein Dok-7 isoform X2
protein Dok-7 isoform X3
protein enabled homolog
protein enabled homolog
protein FAM106A
protein FAM153A
protein FAM153A isoform X1
protein FAM153A isoform X1
protein FAM153A isoform X1
protein FAM153A isoform X1
protein FAM153A isoform X1
protein FAM153A isoform X1
protein FAM153A isoform X1
protein FAM153A isoform X1
protein FAM153A isoform X1
protein FAM153A isoform X1
protein FAM153A isoform X1
protein FAM153A isoform X2
protein FAM153A isoform X3
protein FAM153A isoform X4
protein FAM153A isoform X4
protein FAM153A isoform X5

protein FAM153A isoform X6
protein FAM153A isoform X6
protein FAM153A isoform X6
protein FAM153A isoform X6
protein FAM153A isoform X6
protein FAM153A isoform X6
protein FAM153A isoform X6
protein FAM153A isoform X6
protein FAM153A isoform X6
protein FAM153A isoform X7
protein FAM153B
protein FAM166A isoform X7
protein FAM218A
protein FAM231C
protein FAM231D
protein FAM231D
protein FAM231D
protein FAM231D
protein FAM32A isoform X1
protein FAM89A
protein FAM9A
protein FAM9A
protein GDF5OS, mitochondrial precursor
protein LKAAEAR1 isoform 2
protein LKAAEAR1 isoform X1
protein LKAAEAR1 isoform X1
protein LKAAEAR1 isoform X2
protein PAXX isoform 1
protein PAXX isoform 2
protein phosphatase 1 regulatory subunit 26
protein phosphatase 1 regulatory subunit 26 isoform X1
protein phosphatase 1 regulatory subunit 26 isoform X1
protein phosphatase 1 regulatory subunit 26 isoform X1
protein phosphatase 1 regulatory subunit 26 isoform X1
protein phosphatase 1 regulatory subunit 26 isoform X1
protein phosphatase 1 regulatory subunit 26 isoform X1
protein phosphatase 1 regulatory subunit 26 isoform X1
protein phosphatase 1 regulatory subunit 26 isoform X1
protein phosphatase 1 regulatory subunit 26 isoform X1
protein phosphatase 1 regulatory subunit 26 isoform X1
protein SNORC isoform X1
protein SPT2 homolog
protein STPG3 isoform a
protein STPG3 isoform b
protein STPG3 isoform c

protein STPG3 isoform d
protein STPG3 isoform X1
protein STPG3 isoform X2
protein THEM6 isoform 1
protein THEM6 isoform X1
protein TSSC4 isoform a
protein TSSC4 isoform a
protein TSSC4 isoform a
protein TSSC4 isoform a
protein TSSC4 isoform b
protein TSSC4 isoform X1
protein TSSC4 isoform X1
protein tyrosine phosphatase receptor type C-associated protein precursor
protein ZNF365 isoform D
protein ZNF365 isoform X2
protein ZNF365 isoform X3
protein ZNF365 isoform X4
protein ZNF365 isoform X5
proto-oncogene FRAT1
psoriasis susceptibility 1 candidate gene 1 protein
putative coiled-coil domain-containing protein 144 N-terminal-like isoform X1
putative coiled-coil domain-containing protein 144 N-terminal-like isoform X2
putative coiled-coil domain-containing protein 144 N-terminal-like isoform X3
putative C-_U-editing enzyme APOBEC-4
putative cuticle collagen 91
putative golgin subfamily A member 8I
putative golgin subfamily A member 8I
putative golgin subfamily A member 8I
putative golgin subfamily A member 8I
putative golgin subfamily A member 8I
putative inactive deoxyuridine 5'-triphosphate nucleotidohydrolase-like protein FLJ16323 isoform X1, partial
putative IQ motif and ankyrin repeat domain-containing protein LOC642574
putative IQ motif and ankyrin repeat domain-containing protein LOC642574
putative L-aspartate dehydrogenase isoform 1
putative L-aspartate dehydrogenase isoform 2
putative L-aspartate dehydrogenase isoform X1
putative NPIP-like protein LOC729978
putative NPIP-like protein LOC729978 isoform X1
putative NPIP-like protein LOC729978 isoform X1
putative NPIP-like protein LOC729978 isoform X1
putative NPIP-like protein LOC729978 isoform X2
putative NPIP-like protein LOC729978 isoform X3
putative NPIP-like protein LOC729978 isoform X3
putative NPIP-like protein LOC729978 isoform X4
putative NPIP-like protein LOC729978 isoform X5

putative POM121-like protein 1-like
putative POM121-like protein 1-like
putative POM121-like protein 1-like
putative POM121-like protein 1-like
putative POM121-like protein 1-like
putative POM121-like protein 1-like
putative POM121-like protein 1-like
putative POM121-like protein 1-like
putative POM121-like protein 1-like
putative POM121-like protein 1-like
putative protein FAM106C
putative protein FAM157B isoform X1
putative protein FAM231BP
putative protein MSS51 homolog, mitochondrial
putative uncharacterized protein C10orf126
putative uncharacterized protein C11orf40
putative uncharacterized protein C12orf77 isoform X1
putative uncharacterized protein C15orf56
putative uncharacterized protein C17orf82
putative uncharacterized protein C18orf65
putative uncharacterized protein C19orf81
putative uncharacterized protein C1orf229
putative uncharacterized protein C20orf204 isoform X1
putative uncharacterized protein C20orf78 isoform a
putative uncharacterized protein C22orf34
putative uncharacterized protein C3orf56
putative uncharacterized protein C6orf99 isoform X1
putative uncharacterized protein C6orf99 isoform X2
putative uncharacterized protein C7orf71 isoform a
putative uncharacterized protein C7orf71 isoform X1
putative uncharacterized protein C7orf71 isoform X1
putative uncharacterized protein C7orf71 isoform X2
putative uncharacterized protein C8orf44
putative uncharacterized protein C8orf49
putative uncharacterized protein C9orf62
putative uncharacterized protein encoded by LINC00205
putative uncharacterized protein encoded by LINC00483 isoform X1
putative uncharacterized protein encoded by LINC00483 isoform X1
putative uncharacterized protein encoded by LINC00483 isoform X1
putative uncharacterized protein encoded by LINC00483 isoform X1
putative uncharacterized protein FLJ44672
putative uncharacterized protein FLJ44672
putative uncharacterized protein FLJ44672
putative uncharacterized protein FLJ44672
putative uncharacterized protein FLJ44672

putative uncharacterized protein FLJ44672
putative uncharacterized protein FLJ44672
putative uncharacterized protein FLJ44672
putative uncharacterized protein FLJ44672
putative uncharacterized protein FLJ44672
putative uncharacterized protein FLJ44672
putative uncharacterized protein FLJ44672
putative uncharacterized protein FLJ44672
putative uncharacterized protein FLJ44672 isoform X2
putative uncharacterized protein FLJ44672 isoform X2
putative uncharacterized protein FLJ44672 isoform X3
putative uncharacterized protein FLJ44672, partial
putative uncharacterized protein FLJ44790
putative uncharacterized protein FLJ46204
putative uncharacterized protein FLJ46235
putative uncharacterized protein FLJ92257
putative uncharacterized protein FLJ92257
putative uncharacterized protein LOC388882
putative uncharacterized protein LOC388882
putative uncharacterized protein LOC388882
putative uncharacterized protein LOC388882
putative uncharacterized protein LOC388882
putative uncharacterized protein LOC388882
putative uncharacterized protein LOC388882
putative uncharacterized protein LOC388882
putative uncharacterized protein LOC388882
putative uncharacterized protein LOC388882
putative uncharacterized protein LOC388882
putative uncharacterized protein LOC388882
putative uncharacterized protein LOC388882
putative uncharacterized protein LOC388882
putative uncharacterized protein LOC388882
putative uncharacterized protein LOC388882
putative uncharacterized protein MGC34800
putative uncharacterized protein MGC39545
putative uncharacterized protein UNQ6494/PRO21346
putative uncharacterized protein UNQ9370/PRO34162
putative UPF0607 protein ENSP00000383144
putative UPF0607 protein ENSP00000383783
putative UPF0607 protein FLJ37424
R3H domain-containing protein 4
R3H domain-containing protein 4 isoform X1
rab-like protein 6 isoform 3
ras-related protein Rab-35 isoform X1
ras-related protein Rab-35 isoform X1

reelin domain-containing protein 1 isoform X2
regulator of hemoglobinization and erythroid cell expansion protein
regulator of hemoglobinization and erythroid cell expansion protein isoform X1
repressor of RNA polymerase III transcription MAF1 homolog
repressor of RNA polymerase III transcription MAF1 homolog isoform X1
required for excision 1-B domain-containing protein isoform 1
required for excision 1-B domain-containing protein isoform 2
required for excision 1-B domain-containing protein isoform X1
retbindin isoform 1 precursor
retbindin isoform 1 precursor
retbindin isoform 1 precursor
retbindin isoform 2
retbindin isoform 3
retbindin isoform 4
retbindin isoform 5 precursor
retbindin isoform 6 precursor
retbindin isoform X1
retbindin isoform X2
retbindin isoform X3
retbindin isoform X4
retbindin isoform X5
retbindin isoform X6
retbindin isoform X7
retbindin isoform X8
ribonuclease H2 subunit A
ribonuclease H2 subunit A isoform X1
ribosome biogenesis protein TSR3 homolog
RING finger protein 166 isoform X1
RING finger protein 37 isoform a
RING finger protein 37 isoform b
RING finger protein 37 isoform c
RNA-binding protein 24 isoform X2
RNA-binding protein 3 isoform X1
RNA-binding protein 3 isoform X1
salivary acidic proline-rich phosphoprotein 1/2 isoform a preproprotein
salivary acidic proline-rich phosphoprotein 1/2 isoform b
salivary acidic proline-rich phosphoprotein 1/2 preproprotein
sarcoplasmic reticulum histidine-rich calcium-binding protein isoform X1
sarcoplasmic reticulum histidine-rich calcium-binding protein precursor
secreted and transmembrane protein 1 isoform X1
secreted and transmembrane protein 1 isoform X1
secreted and transmembrane protein 1 precursor
semenogelin-1 preproprotein
semenogelin-2 precursor
serine/arginine repetitive matrix protein 1

serine/arginine repetitive matrix protein 1-like
serine/arginine repetitive matrix protein 2-like isoform X7
serine/arginine-rich splicing factor RSZ22-like
serine/arginine-rich splicing factor RSZ22-like isoform X8
serine palmitoyltransferase small subunit B isoform X1
serine palmitoyltransferase small subunit B isoform X1
serine protease inhibitor Kazal-type 2 isoform X1
serine/threonine-protein kinase 32C isoform X4
serine/threonine-protein kinase haspin
serrate RNA effector molecule homolog isoform a
serrate RNA effector molecule homolog isoform X1
serrate RNA effector molecule homolog isoform X2
serrate RNA effector molecule homolog isoform X3
serrate RNA effector molecule homolog isoform X4
serrate RNA effector molecule homolog isoform X5
serrate RNA effector molecule homolog isoform X6
serrate RNA effector molecule homolog isoform X7
serrate RNA effector molecule homolog isoform X8
SH2 domain-containing protein 6 isoform X13
SH2 domain-containing protein 6 isoform X13
SH2 domain-containing protein 6 isoform X13
SH2 domain-containing protein 6 isoform X14
SH2 domain-containing protein 6 isoform X14
SH2 domain-containing protein 7 isoform X5
sine oculis-binding protein homolog
single-pass membrane and coiled-coil domain-containing protein 4 isoform X1
single-pass membrane and coiled-coil domain-containing protein 4 isoform X2
single-pass membrane and coiled-coil domain-containing protein 4 isoform X3
single-pass membrane and coiled-coil domain-containing protein 4 isoform X4
single-pass membrane and coiled-coil domain-containing protein 4 isoform X5
single-pass membrane and coiled-coil domain-containing protein 4 isoform X5
Sjoegren syndrome/scleroderma autoantigen 1 isoform 1
Sjoegren syndrome/scleroderma autoantigen 1 isoform 2
small integral membrane protein 23
small integral membrane protein 23 isoform X1
small integral membrane protein 27 isoform X1
small integral membrane protein 28
small integral membrane protein 35
small integral membrane protein 35 isoform X1
small integral membrane protein 5 isoform X1
small integral membrane protein 5 isoform X2
snRNA-activating protein complex subunit 4 isoform X3
solute carrier family 22 member 18 isoform c
sperm acrosome-associated protein 7 isoform X5
sperm acrosome-associated protein 7 isoform X7

sperm acrosome-associated protein 7 isoform X8
spermatogenesis-associated protein 2-like protein
spermatogenesis-associated protein 2-like protein isoform X1
sperm protein associated with the nucleus on the X chromosome N4 isoform X1
sperm protein associated with the nucleus on the X chromosome N4 isoform X2
surfeit locus protein 1 isoform 1
surfeit locus protein 1 isoform 2
surfeit locus protein 1 isoform X1
surfeit locus protein 2 isoform 1
surfeit locus protein 2 isoform 2
surfeit locus protein 4 isoform 1
surfeit locus protein 4 isoform 2
surfeit locus protein 4 isoform 3
surfeit locus protein 4 isoform 4
surfeit locus protein 4 isoform X1
surfeit locus protein 4 isoform X1
surfeit locus protein 4 isoform X2
surfeit locus protein 4 isoform X3
surfeit locus protein 6 isoform 1
surfeit locus protein 6 isoform 2
synapsin-1-like
synapsin-1-like
TBC1 domain family member 28 isoform X4
T-cell acute lymphocytic leukemia protein 1 isoform X2
T-complex protein 10A homolog isoform X1
T-complex protein 10A homolog isoform X2
T-complex protein 10A homolog isoform X6
T-complex protein 10A homolog isoform X7
testicular haploid expressed gene protein isoform X1
testicular haploid expressed gene protein isoform X3
testicular haploid expressed gene protein isoform X5
testicular haploid expressed gene protein isoform X6
testis-expressed protein 22 isoform X1
testis-expressed protein 50 isoform X1
tetraspanin-32
tetraspanin-32 isoform X10
tetraspanin-32 isoform X11
tetraspanin-32 isoform X12
tetraspanin-32 isoform X13
tetraspanin-32 isoform X1
tetraspanin-32 isoform X2
tetraspanin-32 isoform X3
tetraspanin-32 isoform X4
tetraspanin-32 isoform X5
tetraspanin-32 isoform X6

tetraspanin-32 isoform X7
tetraspanin-32 isoform X7
tetraspanin-32 isoform X8
tetraspanin-32 isoform X9
thymopoietin isoform X1
TP53 target 3 family member E isoform X1
TP53 target 3 family member E isoform X1
TP53 target 3 family member F isoform X1
TP53 target 3 family member F isoform X1
TP53-target gene 3 protein isoform X1
TP53-target gene 3 protein isoform X1
TP53-target gene 3 protein isoform X1
TP53-target gene 3 protein isoform X1
TP53-target gene 3 protein isoform X1
TP53-target gene 3 protein isoform X1
TP53-target gene 3 protein isoform X1
traB domain-containing protein isoform a
traB domain-containing protein isoform b
traB domain-containing protein isoform b
traB domain-containing protein isoform b
traB domain-containing protein isoform c
traB domain-containing protein isoform X1
traB domain-containing protein isoform X1
traB domain-containing protein isoform X1
traB domain-containing protein isoform X1
traB domain-containing protein isoform X1
traB domain-containing protein isoform X1
traB domain-containing protein isoform X2
traB domain-containing protein isoform X3
traB domain-containing protein isoform X4
traB domain-containing protein isoform X4
traB domain-containing protein isoform X4
traB domain-containing protein isoform X5
transaldolase
transcription factor NF-E4
translation initiation factor IF-2-like
translation initiation factor IF-2-like
translation initiation factor IF-2-like
translation initiation factor IF-2-like
translation initiation factor IF-2-like isoform X1
translation initiation factor IF-2-like isoform X1
transmembrane and immunoglobulin domain containing 3 isoform 4
transmembrane and immunoglobulin domain-containing protein 2 isoform 1 precursor
transmembrane and immunoglobulin domain-containing protein 2 isoform 2 precursor
transmembrane and immunoglobulin domain-containing protein 2 isoform 3

transmembrane and immunoglobulin domain-containing protein 2 isoform X1
transmembrane and immunoglobulin domain-containing protein 2 isoform X2
transmembrane protein 108-like
transmembrane protein 127 isoform X1
transmembrane protein 134 isoform a
transmembrane protein 134 isoform b
transmembrane protein 134 isoform c
transmembrane protein 134 isoform X1
transmembrane protein 134 isoform X2
transmembrane protein 134 isoform X3
transmembrane protein 158 precursor
transmembrane protein 179 isoform X1
transmembrane protein 179 isoform X2
transmembrane protein 179 isoform X3
transmembrane protein 184A isoform X1
transmembrane protein 190 precursor
transmembrane protein 191A
transmembrane protein 205 isoform X1
transmembrane protein 221
transmembrane protein 221 isoform X1
transmembrane protein 225B
transmembrane protein 225B
transmembrane protein 225B
transmembrane protein 225B isoform X1
transmembrane protein 225B isoform X2
transmembrane protein 225B isoform X2
transmembrane protein 225B isoform X2
transmembrane protein 225B isoform X2
transmembrane protein 225B isoform X2
transmembrane protein 225B isoform X2
transmembrane protein 225B isoform X2
transmembrane protein 225B isoform X2
transmembrane protein 225B isoform X3
transmembrane protein 225B isoform X3
transmembrane protein 225B isoform X3
transmembrane protein 225B isoform X3
transmembrane protein 225B isoform X3
transmembrane protein 225B isoform X3
transmembrane protein 241 isoform X6
transmembrane protein 272 isoform 1
transmembrane protein 275
transmembrane protein 31
transmembrane protein 99 isoform X1
transmembrane protein 99 isoform X1
transmembrane protein 99 precursor

transmembrane protein PVRIG
transmembrane protein PVRIG isoform X1
TRIO and F-actin-binding protein-like
TRIO and F-actin-binding protein-like
TRIO and F-actin-binding protein-like
tRNA (adenine(58)-N(1))-methyltransferase, mitochondrial isoform X5
tRNA modification GTPase GTPBP3, mitochondrial isoform IV
tRNA modification GTPase GTPBP3, mitochondrial isoform V
tRNA modification GTPase GTPBP3, mitochondrial isoform VII
tRNA pseudouridine synthase-like 1 isoform X1
tRNA pseudouridine synthase-like 1 isoform X2
tRNA-splicing endonuclease subunit Sen34 isoform 1
tRNA-splicing endonuclease subunit Sen34 isoform 1
tRNA-splicing endonuclease subunit Sen34 isoform 1
tRNA-splicing endonuclease subunit Sen34 isoform 2
tRNA-splicing endonuclease subunit Sen34 isoform X1
tRNA-splicing endonuclease subunit Sen34 isoform X2
tumor necrosis factor alpha-induced protein 2 isoform X3
tumor necrosis factor ligand superfamily member 9
tumor necrosis factor receptor superfamily member 14 isoform X1
tumor necrosis factor receptor superfamily member 14 isoform X2
tumor necrosis factor receptor superfamily member 18 isoform 1 precursor
tumor necrosis factor receptor superfamily member 18 isoform 2 precursor
tumor necrosis factor receptor superfamily member 18 isoform 3 precursor
tumor necrosis factor receptor superfamily member 18 isoform X1
tumor necrosis factor receptor superfamily member 4 isoform X1
tumor necrosis factor receptor superfamily member 4 isoform X3
tumor necrosis factor receptor superfamily member 4 isoform X5
type-1 angiotensin II receptor-associated protein isoform X2
U4/U6.U5 tri-snRNP-associated protein 1 isoform X1
U4/U6.U5 tri-snRNP-associated protein 1 isoform X2
ubiquitin-conjugating enzyme E2 E2 isoform X3
ubiquitin-conjugating enzyme E2 E2 isoform X4
ubiquitin-conjugating enzyme E2 E2 isoform X5
UDP-GalNAc:beta-1,3-N-acetylgalactosaminyltransferase 1 isoform X2
uncharacterized protein C10orf111
uncharacterized protein C10orf143 isoform X1
uncharacterized protein C10orf143 isoform X2
uncharacterized protein C10orf143 isoform X3
uncharacterized protein C10orf55
uncharacterized protein C10orf95
uncharacterized protein C11orf21 isoform X1
uncharacterized protein C13orf42
uncharacterized protein C13orf42 isoform X1
uncharacterized protein C13orf46 isoform X2

uncharacterized protein C13orf46 isoform X2
uncharacterized protein C13orf46 isoform X3
uncharacterized protein C13orf46 isoform X3
uncharacterized protein C13orf46 isoform X4
uncharacterized protein C13orf46 isoform X4
uncharacterized protein C13orf46 isoform X5
uncharacterized protein C13orf46 isoform X5
uncharacterized protein C13orf46 isoform X6
uncharacterized protein C13orf46 isoform X6
uncharacterized protein C13orf46 isoform X7
uncharacterized protein C13orf46 isoform X8
uncharacterized protein C13orf46 isoform X9
uncharacterized protein C15orf32 isoform a
uncharacterized protein C15orf32 isoform X1
uncharacterized protein C15orf53
uncharacterized protein C16orf92 isoform 3 precursor
uncharacterized protein C17orf102
uncharacterized protein C17orf51
uncharacterized protein C17orf51 isoform X1
uncharacterized protein C17orf51 isoform X1
uncharacterized protein C17orf51 isoform X1
uncharacterized protein C17orf77
uncharacterized protein C17orf77
uncharacterized protein C18orf12
uncharacterized protein C19orf57 isoform 1
uncharacterized protein C19orf57 isoform 2
uncharacterized protein C19orf57 isoform 3
uncharacterized protein C19orf57 isoform 4
uncharacterized protein C19orf57 isoform 5
uncharacterized protein C19orf57 isoform 6
uncharacterized protein C19orf57 isoform X1
uncharacterized protein C19orf57 isoform X2
uncharacterized protein C19orf57 isoform X3
uncharacterized protein C19orf57 isoform X4
uncharacterized protein C19orf57 isoform X5
uncharacterized protein C19orf57 isoform X5
uncharacterized protein C19orf57 isoform X6
uncharacterized protein C19orf57 isoform X6
uncharacterized protein C19orf57 isoform X7
uncharacterized protein C19orf71
uncharacterized protein C19orf71 isoform X1
uncharacterized protein C1orf159 isoform 1 precursor
uncharacterized protein C1orf159 isoform 2 precursor
uncharacterized protein C1orf159 isoform X1
uncharacterized protein C1orf159 isoform X1

uncharacterized protein C1orf159 isoform X1
uncharacterized protein C1orf159 isoform X1
uncharacterized protein C1orf159 isoform X1
uncharacterized protein C1orf159 isoform X1
uncharacterized protein C1orf159 isoform X1
uncharacterized protein C1orf159 isoform X1
uncharacterized protein C1orf159 isoform X1
uncharacterized protein C1orf159 isoform X1
uncharacterized protein C1orf159 isoform X1
uncharacterized protein C1orf159 isoform X1
uncharacterized protein C1orf159 isoform X2
uncharacterized protein C1orf159 isoform X2
uncharacterized protein C1orf159 isoform X2
uncharacterized protein C1orf159 isoform X3
uncharacterized protein C1orf159 isoform X3
uncharacterized protein C1orf159 isoform X4
uncharacterized protein C1orf159 isoform X4
uncharacterized protein C1orf159 isoform X4
uncharacterized protein C1orf159 isoform X4
uncharacterized protein C1orf159 isoform X4
uncharacterized protein C1orf159 isoform X4
uncharacterized protein C1orf159 isoform X4
uncharacterized protein C1orf159 isoform X4
uncharacterized protein C1orf159 isoform X4
uncharacterized protein C1orf159 isoform X5
uncharacterized protein C1orf159 isoform X6
uncharacterized protein C20orf173 isoform X1
uncharacterized protein C20orf173 isoform X2
uncharacterized protein C20orf173 isoform X3
uncharacterized protein C20orf173 precursor
uncharacterized protein C20orf203
uncharacterized protein C20orf203 isoform X1
uncharacterized protein C22orf15 isoform X11
uncharacterized protein C22orf15 isoform X15
uncharacterized protein C22orf15 isoform X16
uncharacterized protein C22orf15 isoform X17
uncharacterized protein C22orf15 isoform X1
uncharacterized protein C22orf15 isoform X3
uncharacterized protein C22orf24 isoform a
uncharacterized protein C22orf24 isoform c
uncharacterized protein C22orf34 isoform X1
uncharacterized protein C22orf42
uncharacterized protein C22orf42 isoform X1
uncharacterized protein C22orf42 isoform X2
uncharacterized protein C22orf42 isoform X3

uncharacterized protein C22orf42 isoform X4
uncharacterized protein C22orf42 isoform X5
uncharacterized protein C2orf27A isoform 1
uncharacterized protein C2orf27A isoform 1
uncharacterized protein C2orf27B
uncharacterized protein C2orf48
uncharacterized protein C2orf50
uncharacterized protein C2orf50 isoform X1
uncharacterized protein C2orf50 isoform X1
uncharacterized protein C2orf50 isoform X1
uncharacterized protein C2orf91 isoform X1
uncharacterized protein C3orf18 isoform X3
uncharacterized protein C3orf18 isoform X3
uncharacterized protein C3orf18 isoform X3
uncharacterized protein C3orf18 isoform X3
uncharacterized protein C3orf18 isoform X3
uncharacterized protein C3orf18 isoform X3
uncharacterized protein C3orf18 isoform X3
uncharacterized protein C3orf36
uncharacterized protein C6orf223 isoform 1
uncharacterized protein C6orf223 isoform X1
uncharacterized protein C7orf33
uncharacterized protein C7orf50 isoform a
uncharacterized protein C7orf50 isoform a
uncharacterized protein C7orf50 isoform a
uncharacterized protein C7orf50 isoform a
uncharacterized protein C7orf50 isoform b
uncharacterized protein C7orf50 isoform c
uncharacterized protein C7orf50 isoform X1
uncharacterized protein C7orf50 isoform X2
uncharacterized protein C7orf50 isoform X3
uncharacterized protein C7orf50 isoform X3
uncharacterized protein C7orf50 isoform X3
uncharacterized protein C7orf50 isoform X3
uncharacterized protein C7orf50 isoform X3
uncharacterized protein C7orf50 isoform X4
uncharacterized protein C7orf50 isoform X5
uncharacterized protein C7orf50 isoform X6
uncharacterized protein C7orf65
uncharacterized protein C8orf86 isoform 1
uncharacterized protein C9orf139
uncharacterized protein C9orf139 isoform X1
uncharacterized protein C9orf163
uncharacterized protein C9orf47 isoform 1 precursor
uncharacterized protein C9orf47 isoform 2 precursor

uncharacterized protein C9orf50
uncharacterized protein C9orf50 isoform X1
uncharacterized protein C9orf50 isoform X1
uncharacterized protein C9orf50 isoform X2
uncharacterized protein C9orf50 isoform X3
uncharacterized protein C9orf50 isoform X4
uncharacterized protein C9orf50 isoform X4
uncharacterized protein C9orf50 isoform X5
uncharacterized protein C9orf50 isoform X6
uncharacterized protein C9orf50 isoform X7
uncharacterized protein C9orf50 isoform X8
uncharacterized protein C9orf66
uncharacterized protein CH507-42P11.6
uncharacterized protein CH507-42P11.6
uncharacterized protein CH507-42P11.6
uncharacterized protein CLBA1 isoform 1
uncharacterized protein CLBA1 isoform X1
uncharacterized protein CLBA1 isoform X2
uncharacterized protein ENSP00000382033
uncharacterized protein ENSP00000382042
uncharacterized protein ENSP00000382042-like
uncharacterized protein FAM120AOS isoform 1
uncharacterized protein FLJ20712
uncharacterized protein FLJ40521 isoform X1
uncharacterized protein FLJ40521 isoform X2
uncharacterized protein FLJ40521 isoform X3
uncharacterized protein FLJ40521-like
uncharacterized protein FLJ40521-like
uncharacterized protein FLJ46347-like
uncharacterized protein GATD3B isoform X5
uncharacterized protein KIAA0040 isoform X1
uncharacterized protein KIAA0040 isoform X1
uncharacterized protein KIAA0040 isoform X1
uncharacterized protein KIAA0040 isoform X1
uncharacterized protein KIAA0040 isoform X1
uncharacterized protein KIAA0040 isoform X1
uncharacterized protein KIAA0040 isoform X1
uncharacterized protein LOC100128124
uncharacterized protein LOC100129098
uncharacterized protein LOC100129697
uncharacterized protein LOC100129940 isoform 1
uncharacterized protein LOC100129940 isoform 2
uncharacterized protein LOC100130357
uncharacterized protein LOC100130370 isoform X1
uncharacterized protein LOC100130370 isoform X2

uncharacterized protein LOC100130370 isoform X3
uncharacterized protein LOC100130370 isoform X4
uncharacterized protein LOC100130449
uncharacterized protein LOC100131094
uncharacterized protein LOC100132874
uncharacterized protein LOC100133204
uncharacterized protein LOC100134391
uncharacterized protein LOC100134391 isoform X1
uncharacterized protein LOC100286986
uncharacterized protein LOC100506571
uncharacterized protein LOC100507221
uncharacterized protein LOC100507507
uncharacterized protein LOC100652901
uncharacterized protein LOC100996259
uncharacterized protein LOC100996274
uncharacterized protein LOC100996318
uncharacterized protein LOC100996413
uncharacterized protein LOC100996413
uncharacterized protein LOC100996574
uncharacterized protein LOC100996598
uncharacterized protein LOC100996701
uncharacterized protein LOC100996720
uncharacterized protein LOC100996842 isoform 1
uncharacterized protein LOC100996842 isoform X1
uncharacterized protein LOC101060017
uncharacterized protein LOC101060017
uncharacterized protein LOC101060179
uncharacterized protein LOC101060341 isoform X1
uncharacterized protein LOC101060341 isoform X1
uncharacterized protein LOC101060588
uncharacterized protein LOC101060588
uncharacterized protein LOC101060588
uncharacterized protein LOC101927353
uncharacterized protein LOC101927353
uncharacterized protein LOC101927353
uncharacterized protein LOC101927353
uncharacterized protein LOC101927353
uncharacterized protein LOC101927353
uncharacterized protein LOC101927353
uncharacterized protein LOC101927375 isoform X1
uncharacterized protein LOC101927375 isoform X1
uncharacterized protein LOC101927375 isoform X1
uncharacterized protein LOC101927401 isoform X1
uncharacterized protein LOC101927503 isoform X1
uncharacterized protein LOC101927503 isoform X1

uncharacterized protein LOC101927562
uncharacterized protein LOC101927824
uncharacterized protein LOC101927824
uncharacterized protein LOC101927824
uncharacterized protein LOC101928095 isoform X1
uncharacterized protein LOC101928095 isoform X1
uncharacterized protein LOC101928095 isoform X1
uncharacterized protein LOC101928095 isoform X1
uncharacterized protein LOC101928095 isoform X2
uncharacterized protein LOC101928095 isoform X3
uncharacterized protein LOC101928095 isoform X4
uncharacterized protein LOC101928095 isoform X5
uncharacterized protein LOC101928095 isoform X6
uncharacterized protein LOC101928120
uncharacterized protein LOC101928193 isoform X1
uncharacterized protein LOC101928193 isoform X2
uncharacterized protein LOC101928193 isoform X3
uncharacterized protein LOC101928212 isoform X1
uncharacterized protein LOC101928212 isoform X2
uncharacterized protein LOC101928268
uncharacterized protein LOC101928548
uncharacterized protein LOC101928764 isoform X2
uncharacterized protein LOC101929097
uncharacterized protein LOC101929372
uncharacterized protein LOC101929400 isoform X1
uncharacterized protein LOC101929400 isoform X2
uncharacterized protein LOC101929400 isoform X3
uncharacterized protein LOC101929692
uncharacterized protein LOC101929747
uncharacterized protein LOC101929895
uncharacterized protein LOC101929937
uncharacterized protein LOC101930307 isoform X10
uncharacterized protein LOC101930307 isoform X11
uncharacterized protein LOC101930307 isoform X12
uncharacterized protein LOC101930307 isoform X13
uncharacterized protein LOC101930307 isoform X14
uncharacterized protein LOC101930307 isoform X15
uncharacterized protein LOC101930307 isoform X16
uncharacterized protein LOC101930307 isoform X17
uncharacterized protein LOC101930307 isoform X18
uncharacterized protein LOC101930307 isoform X19
uncharacterized protein LOC101930307 isoform X1
uncharacterized protein LOC101930307 isoform X20
uncharacterized protein LOC101930307 isoform X21
uncharacterized protein LOC101930307 isoform X22

uncharacterized protein LOC101930307 isoform X23
uncharacterized protein LOC101930307 isoform X24
uncharacterized protein LOC101930307 isoform X25
uncharacterized protein LOC101930307 isoform X26
uncharacterized protein LOC101930307 isoform X26
uncharacterized protein LOC101930307 isoform X27
uncharacterized protein LOC101930307 isoform X28
uncharacterized protein LOC101930307 isoform X29
uncharacterized protein LOC101930307 isoform X2
uncharacterized protein LOC101930307 isoform X3
uncharacterized protein LOC101930307 isoform X4
uncharacterized protein LOC101930307 isoform X5
uncharacterized protein LOC101930307 isoform X6
uncharacterized protein LOC101930307 isoform X7
uncharacterized protein LOC101930307 isoform X8
uncharacterized protein LOC101930307 isoform X9
uncharacterized protein LOC102723360 isoform X1
uncharacterized protein LOC102723750 isoform X1
uncharacterized protein LOC102723750 isoform X1
uncharacterized protein LOC102723750 isoform X1
uncharacterized protein LOC102723750 isoform X1
uncharacterized protein LOC102723750 isoform X1
uncharacterized protein LOC102723750 isoform X1
uncharacterized protein LOC102723750 isoform X1
uncharacterized protein LOC102723750 isoform X2
uncharacterized protein LOC102723750 isoform X2
uncharacterized protein LOC102723750 isoform X3
uncharacterized protein LOC102724014, partial
uncharacterized protein LOC102724052, partial
uncharacterized protein LOC102724062
uncharacterized protein LOC102724219 isoform X1
uncharacterized protein LOC102724219 isoform X1
uncharacterized protein LOC102724378 isoform X1
uncharacterized protein LOC102724646
uncharacterized protein LOC102724657 isoform X1
uncharacterized protein LOC102724657 isoform X2
uncharacterized protein LOC102724720 isoform X1
uncharacterized protein LOC102724720 isoform X2
uncharacterized protein LOC102724843 isoform X1
uncharacterized protein LOC102724843 isoform X1
uncharacterized protein LOC102724843 isoform X1
uncharacterized protein LOC102724877 isoform X1
uncharacterized protein LOC102724877 isoform X2
uncharacterized protein LOC102724877 isoform X3
uncharacterized protein LOC102724877 isoform X4

uncharacterized protein LOC102724951 isoform X1
uncharacterized protein LOC102724951 isoform X1
uncharacterized protein LOC102724965 isoform X1
uncharacterized protein LOC102724965 isoform X2
uncharacterized protein LOC105369201 isoform X1
uncharacterized protein LOC105369201 isoform X1
uncharacterized protein LOC105369201 isoform X2
uncharacterized protein LOC105369201 isoform X3
uncharacterized protein LOC105369201 isoform X4
uncharacterized protein LOC105369201 isoform X5
uncharacterized protein LOC105369205 isoform X3
uncharacterized protein LOC105369214
uncharacterized protein LOC105369246
uncharacterized protein LOC105369266
uncharacterized protein LOC105369535 isoform X1
uncharacterized protein LOC105369535 isoform X1
uncharacterized protein LOC105369535 isoform X2
uncharacterized protein LOC105369535 isoform X3
uncharacterized protein LOC105369535 isoform X4
uncharacterized protein LOC105369535 isoform X5
uncharacterized protein LOC105369535 isoform X6
uncharacterized protein LOC105369591 isoform X1
uncharacterized protein LOC105369591 isoform X2
uncharacterized protein LOC105369869
uncharacterized protein LOC105369914
uncharacterized protein LOC105370092
uncharacterized protein LOC105370295
uncharacterized protein LOC105370362 isoform X1
uncharacterized protein LOC105370362 isoform X2
uncharacterized protein LOC105370399 isoform X1
uncharacterized protein LOC105370399 isoform X2
uncharacterized protein LOC105370579
uncharacterized protein LOC105370641
uncharacterized protein LOC105370687
uncharacterized protein LOC105370691
uncharacterized protein LOC105370706
uncharacterized protein LOC105370708
uncharacterized protein LOC105370733 isoform X1
uncharacterized protein LOC105370733 isoform X2
uncharacterized protein LOC105370733 isoform X3
uncharacterized protein LOC105370940
uncharacterized protein LOC105370940
uncharacterized protein LOC105370940
uncharacterized protein LOC105371031
uncharacterized protein LOC105371045

uncharacterized protein LOC105371191
uncharacterized protein LOC105371253
uncharacterized protein LOC105371371
uncharacterized protein LOC105371419
uncharacterized protein LOC105371566
uncharacterized protein LOC105371598
uncharacterized protein LOC105371763
uncharacterized protein LOC105371763
uncharacterized protein LOC105371910
uncharacterized protein LOC105371921 isoform X1
uncharacterized protein LOC105371921 isoform X2
uncharacterized protein LOC105371930
uncharacterized protein LOC105371932
uncharacterized protein LOC105371933
uncharacterized protein LOC105371944 isoform X1
uncharacterized protein LOC105371944 isoform X2
uncharacterized protein LOC105372109
uncharacterized protein LOC105372204
uncharacterized protein LOC105372585
uncharacterized protein LOC105372704
uncharacterized protein LOC105372704
uncharacterized protein LOC105372714 isoform X1
uncharacterized protein LOC105372714 isoform X2
uncharacterized protein LOC105372798
uncharacterized protein LOC105372824
uncharacterized protein LOC105372836
uncharacterized protein LOC105372883 isoform X1
uncharacterized protein LOC105372883 isoform X2
uncharacterized protein LOC105373102
uncharacterized protein LOC105373102
uncharacterized protein LOC105373132
uncharacterized protein LOC105373132
uncharacterized protein LOC105373133
uncharacterized protein LOC105373242 isoform X1
uncharacterized protein LOC105373242 isoform X1
uncharacterized protein LOC105373242 isoform X1
uncharacterized protein LOC105373242 isoform X2
uncharacterized protein LOC105373244 isoform X1
uncharacterized protein LOC105373244 isoform X2
uncharacterized protein LOC105373311
uncharacterized protein LOC105373347 isoform X1
uncharacterized protein LOC105373347 isoform X1
uncharacterized protein LOC105373347 isoform X1
uncharacterized protein LOC105373347 isoform X2
uncharacterized protein LOC105373392

uncharacterized protein LOC105373392
uncharacterized protein LOC105373395 isoform X1
uncharacterized protein LOC105373395 isoform X2
uncharacterized protein LOC105373750
uncharacterized protein LOC105373759
uncharacterized protein LOC105373944
uncharacterized protein LOC105374089
uncharacterized protein LOC105374250 isoform 1
uncharacterized protein LOC105374250 isoform X1
uncharacterized protein LOC105374250 isoform X2
uncharacterized protein LOC105374299
uncharacterized protein LOC105374299
uncharacterized protein LOC105374299
uncharacterized protein LOC105374314
uncharacterized protein LOC105374378 isoform X1
uncharacterized protein LOC105374378 isoform X2
uncharacterized protein LOC105374378 isoform X3
uncharacterized protein LOC105374378 isoform X4
uncharacterized protein LOC105374378 isoform X5
uncharacterized protein LOC105374378 isoform X6
uncharacterized protein LOC105374378 isoform X7
uncharacterized protein LOC105374378 isoform X8
uncharacterized protein LOC105374811 isoform X1
uncharacterized protein LOC105374811 isoform X2
uncharacterized protein LOC105375106
uncharacterized protein LOC105375107
uncharacterized protein LOC105375303
uncharacterized protein LOC105375683
uncharacterized protein LOC105375938 isoform X1
uncharacterized protein LOC105375938 isoform X2
uncharacterized protein LOC105376204
uncharacterized protein LOC105376341
uncharacterized protein LOC105376353 isoform X1
uncharacterized protein LOC105376684
uncharacterized protein LOC105376714
uncharacterized protein LOC105376791 isoform X1
uncharacterized protein LOC105376791 isoform X2
uncharacterized protein LOC105376875
uncharacterized protein LOC105376875
uncharacterized protein LOC105376875
uncharacterized protein LOC105376917 isoform X1
uncharacterized protein LOC105377021
uncharacterized protein LOC105377022
uncharacterized protein LOC105377622
uncharacterized protein LOC105377650

uncharacterized protein LOC105377746
uncharacterized protein LOC105377777 isoform X1
uncharacterized protein LOC105377777 isoform X1
uncharacterized protein LOC105377777 isoform X2
uncharacterized protein LOC105378105
uncharacterized protein LOC105378148 isoform X1
uncharacterized protein LOC105378148 isoform X2
uncharacterized protein LOC105378161
uncharacterized protein LOC105378161
uncharacterized protein LOC105378189
uncharacterized protein LOC105378479
uncharacterized protein LOC105378592
uncharacterized protein LOC105378592
uncharacterized protein LOC105378696 isoform X1
uncharacterized protein LOC105378696 isoform X1
uncharacterized protein LOC105379177
uncharacterized protein LOC105379198
uncharacterized protein LOC105379473
uncharacterized protein LOC105379474
uncharacterized protein LOC105379533
uncharacterized protein LOC105379545
uncharacterized protein LOC105379561 isoform X1
uncharacterized protein LOC105379561 isoform X2
uncharacterized protein LOC105379561 isoform X3
uncharacterized protein LOC105379561 isoform X4
uncharacterized protein LOC107983958
uncharacterized protein LOC107983981 isoform X1
uncharacterized protein LOC107983981 isoform X1
uncharacterized protein LOC107983981 isoform X2
uncharacterized protein LOC107983981 isoform X3
uncharacterized protein LOC107983989
uncharacterized protein LOC107983998 isoform X1
uncharacterized protein LOC107983998 isoform X2
uncharacterized protein LOC107984104
uncharacterized protein LOC107984104
uncharacterized protein LOC107984104
uncharacterized protein LOC107984104
uncharacterized protein LOC107984104
uncharacterized protein LOC107984159
uncharacterized protein LOC107984236
uncharacterized protein LOC107984265
uncharacterized protein LOC107984282
uncharacterized protein LOC107984282
uncharacterized protein LOC107984282
uncharacterized protein LOC107984341

uncharacterized protein LOC107984399
uncharacterized protein LOC107984449
uncharacterized protein LOC107984449
uncharacterized protein LOC107984449
uncharacterized protein LOC107984449
uncharacterized protein LOC107984449
uncharacterized protein LOC107984449
uncharacterized protein LOC107984512
uncharacterized protein LOC107984648
uncharacterized protein LOC107984728
uncharacterized protein LOC107984751
uncharacterized protein LOC107984751
uncharacterized protein LOC107984813
uncharacterized protein LOC107984814 isoform X1
uncharacterized protein LOC107984817
uncharacterized protein LOC107984820
uncharacterized protein LOC107984832 isoform X1
uncharacterized protein LOC107984833
uncharacterized protein LOC107984851
uncharacterized protein LOC107984859 isoform X1
uncharacterized protein LOC107984859 isoform X2
uncharacterized protein LOC107984859 isoform X3
uncharacterized protein LOC107984859 isoform X4
uncharacterized protein LOC107984859 isoform X5
uncharacterized protein LOC107984862
uncharacterized protein LOC107984876
uncharacterized protein LOC107985022
uncharacterized protein LOC107985103
uncharacterized protein LOC107985115
uncharacterized protein LOC107985246
uncharacterized protein LOC107985320 isoform X1
uncharacterized protein LOC107985320 isoform X1
uncharacterized protein LOC107985320 isoform X2
uncharacterized protein LOC107985381
uncharacterized protein LOC107985384
uncharacterized protein LOC107985410
uncharacterized protein LOC107985416 isoform X1
uncharacterized protein LOC107985416 isoform X2
uncharacterized protein LOC107985428
uncharacterized protein LOC107985535
uncharacterized protein LOC107985678
uncharacterized protein LOC107985687
uncharacterized protein LOC107985729
uncharacterized protein LOC107985734
uncharacterized protein LOC107985770 isoform X2

uncharacterized protein LOC107985773
uncharacterized protein LOC107985792
uncharacterized protein LOC107985795
uncharacterized protein LOC107985803
uncharacterized protein LOC107985856 isoform X1
uncharacterized protein LOC107985856 isoform X2
uncharacterized protein LOC107985876
uncharacterized protein LOC107985876
uncharacterized protein LOC107985896
uncharacterized protein LOC107985911
uncharacterized protein LOC107985939
uncharacterized protein LOC107985946
uncharacterized protein LOC107986004
uncharacterized protein LOC107986004
uncharacterized protein LOC107986163
uncharacterized protein LOC107986175
uncharacterized protein LOC107986192
uncharacterized protein LOC107986211
uncharacterized protein LOC107986215
uncharacterized protein LOC107986255
uncharacterized protein LOC107986352
uncharacterized protein LOC107986353
uncharacterized protein LOC107986354
uncharacterized protein LOC107986531
uncharacterized protein LOC107986554
uncharacterized protein LOC107986554
uncharacterized protein LOC107986554
uncharacterized protein LOC107986554
uncharacterized protein LOC107986554
uncharacterized protein LOC107986755
uncharacterized protein LOC107986755
uncharacterized protein LOC107986762
uncharacterized protein LOC107986762
uncharacterized protein LOC107986777 isoform X1
uncharacterized protein LOC107986777 isoform X2
uncharacterized protein LOC107986791
uncharacterized protein LOC107986791
uncharacterized protein LOC107986791
uncharacterized protein LOC107986791
uncharacterized protein LOC107986797
uncharacterized protein LOC107986800 isoform X1
uncharacterized protein LOC107986810
uncharacterized protein LOC107986908
uncharacterized protein LOC107986910 isoform X1
uncharacterized protein LOC107986910 isoform X1

uncharacterized protein LOC107986910 isoform X2
uncharacterized protein LOC107987044
uncharacterized protein LOC107987096
uncharacterized protein LOC107987125
uncharacterized protein LOC107987142
uncharacterized protein LOC107987158
uncharacterized protein LOC107987158
uncharacterized protein LOC107987158
uncharacterized protein LOC107987158
uncharacterized protein LOC107987188
uncharacterized protein LOC107987211
uncharacterized protein LOC107987211
uncharacterized protein LOC107987233
uncharacterized protein LOC107987237
uncharacterized protein LOC107987243
uncharacterized protein LOC107987269
uncharacterized protein LOC107987276
uncharacterized protein LOC107987285
uncharacterized protein LOC107987288
uncharacterized protein LOC107987345
uncharacterized protein LOC107987363
uncharacterized protein LOC107987372
uncharacterized protein LOC107987377
uncharacterized protein LOC107987386
uncharacterized protein LOC107987388 isoform X1
uncharacterized protein LOC107987388 isoform X2
uncharacterized protein LOC107987388 isoform X2
uncharacterized protein LOC107987394
uncharacterized protein LOC107987469
uncharacterized protein LOC112267857 isoform X1
uncharacterized protein LOC112267874
uncharacterized protein LOC112267876
uncharacterized protein LOC112267930
uncharacterized protein LOC112267934
uncharacterized protein LOC112267939
uncharacterized protein LOC112267940
uncharacterized protein LOC112267947 isoform X1
uncharacterized protein LOC112267971
uncharacterized protein LOC112267991
uncharacterized protein LOC112268013
uncharacterized protein LOC112268052
uncharacterized protein LOC112268068
uncharacterized protein LOC112268076
uncharacterized protein LOC112268102
uncharacterized protein LOC112268105

uncharacterized protein LOC112268131 isoform X1
uncharacterized protein LOC112268131 isoform X2
uncharacterized protein LOC112268136
uncharacterized protein LOC112268145
uncharacterized protein LOC112268184
uncharacterized protein LOC112268198
uncharacterized protein LOC112268219
uncharacterized protein LOC112268232
uncharacterized protein LOC112268233
uncharacterized protein LOC112268237
uncharacterized protein LOC112268260 isoform X1
uncharacterized protein LOC112268260 isoform X2
uncharacterized protein LOC112268271
uncharacterized protein LOC112268305
uncharacterized protein LOC112268321
uncharacterized protein LOC112268321
uncharacterized protein LOC112268349
uncharacterized protein LOC112268350
uncharacterized protein LOC112268394
uncharacterized protein LOC112268398
uncharacterized protein LOC112268414
uncharacterized protein LOC112268444
uncharacterized protein LOC112268452
uncharacterized protein LOC112268453
uncharacterized protein LOC112268459
uncharacterized protein LOC285500 isoform X1
uncharacterized protein LOC285500 isoform X2
uncharacterized protein LOC388282
uncharacterized protein LOC388282 isoform X1
uncharacterized protein LOC388780 isoform X1
uncharacterized protein LOC388780 isoform X1
uncharacterized protein LOC388780 isoform X2
uncharacterized protein LOC389199
uncharacterized protein LOC389602
uncharacterized protein LOC389602 isoform X1
uncharacterized protein LOC401040
uncharacterized protein LOC401478 isoform X1
uncharacterized protein LOC401478 isoform X1
uncharacterized protein LOC401478 isoform X2
uncharacterized protein LOC401478 isoform X3
uncharacterized protein LOC401478 isoform X4
uncharacterized protein LOC401478 isoform X5
uncharacterized protein LOC401478 precursor
uncharacterized protein LOC441239
uncharacterized protein LOC642249

uncharacterized protein LOC643365
uncharacterized protein LOC643365 isoform X1
uncharacterized protein LOC644090 isoform X1
uncharacterized protein LOC645188
uncharacterized protein LOC732265
uncharacterized protein SMIM29 isoform 1 precursor
uncharacterized protein SMIM29 isoform 1 precursor
uncharacterized protein SMIM29 isoform 1 precursor
uncharacterized protein SMIM29 isoform 1 precursor
UPF0573 protein C2orf70 isoform 2
UPF0573 protein C2orf70 isoform X5
UPF0598 protein C8orf82
Usher syndrome type-1C protein-binding protein 1 isoform X1
UV-stimulated scaffold protein A isoform X3
UV-stimulated scaffold protein A isoform X4
UV-stimulated scaffold protein A isoform X5
UV-stimulated scaffold protein A isoform X6
variable charge X-linked protein 1
variable charge X-linked protein 1 isoform X1
variable charge X-linked protein 3
vasohibin-2 isoform X7
vegetative cell wall protein gp1-like
vegetative cell wall protein gp1-like
vegetative cell wall protein gp1-like
vegetative cell wall protein gp1-like
vegetative cell wall protein gp1-like isoform X1
vegetative cell wall protein gp1-like isoform X2
vesicle transport protein USE1
vesicle transport protein USE1 isoform X1
vesicular, overexpressed in cancer, prosurvival protein 1 isoform 11 precursor
vesicular, overexpressed in cancer, prosurvival protein 1 isoform 11 precursor
vesicular, overexpressed in cancer, prosurvival protein 1 isoform 11 precursor
vesicular, overexpressed in cancer, prosurvival protein 1 isoform 5
vesicular, overexpressed in cancer, prosurvival protein 1 isoform 6 precursor
vimentin-type intermediate filament-associated coiled-coil protein
voltage-dependent calcium channel beta subunit-associated regulatory protein
voltage-dependent calcium channel beta subunit-associated regulatory protein isoform X1
voltage-dependent calcium channel beta subunit-associated regulatory protein isoform X2
voltage-dependent calcium channel beta subunit-associated regulatory protein isoform X3
voltage-dependent calcium channel beta subunit-associated regulatory protein isoform X3
voltage-dependent calcium channel beta subunit-associated regulatory protein isoform X4
voltage-dependent calcium channel beta subunit-associated regulatory protein isoform X5
voltage-dependent calcium channel beta subunit-associated regulatory protein isoform X6
voltage-dependent calcium channel beta subunit-associated regulatory protein isoform X6
V-set and immunoglobulin domain-containing protein 10-like 2 isoform X2

V-set and immunoglobulin domain-containing protein 10-like 2 isoform X3
WAS/WASL-interacting protein family member 2-like
WAS/WASL-interacting protein family member 3-like
WD repeat-containing protein 93 isoform X4
WD repeat-containing protein 93 isoform X5
WD repeat-containing protein 93 isoform X5
WD repeat-containing protein 93 isoform X5
WD repeat-containing protein 93 isoform X5
WD repeat-containing protein WRAP73 isoform X2
WNT1-inducible-signaling pathway protein 2 isoform X1
WW domain-containing oxidoreductase isoform X6
XIAP-associated factor 1 isoform X5
yjeF N-terminal domain-containing protein 3 isoform X3
YJU2 splicing factor homolog
Z-DNA-binding protein 1 isoform a
Z-DNA-binding protein 1 isoform b
Z-DNA-binding protein 1 isoform c
Z-DNA-binding protein 1 isoform d
Z-DNA-binding protein 1 isoform e
Z-DNA-binding protein 1 isoform X1
Z-DNA-binding protein 1 isoform X2
Z-DNA-binding protein 1 isoform X3
zinc finger protein 586 isoform b
zinc finger protein 74 isoform b
zinc finger protein 778 isoform X4
zinc finger protein 778 isoform X5
zinc finger protein 778 isoform X5
zinc finger protein 778 isoform X5
zinc finger protein 778 isoform X6
zinc finger protein 843
zinc finger protein 843
ZO-2 associated speckle protein

Unique human protein sequences missing in mouse
14-3-3 protein zeta/delta isoform X2
14-3-3 protein zeta/delta isoform X3
36.4 kDa proline-rich protein
36.4 kDa proline-rich protein
acyl-CoA synthetase family member 3, mitochondrial isoform X3
adhesive plaque matrix protein-like
alpha-2,8-sialyltransferase 8F isoform X2
ankyrin repeat domain-containing protein 20A2-like
ankyrin repeat domain-containing protein 20B-like
anthrax toxin receptor-like isoform 2
anthrax toxin receptor-like isoform X9
AP20 region protein 1 isoform B
arf-GAP with GTPase, ANK repeat and PH domain-containing protein 2
arf-GAP with GTPase, ANK repeat and PH domain-containing protein 2-like
aspartate-rich protein 1
aspartate-rich protein 1 isoform X1
aspartate-rich protein 1 isoform X2
aspartate-rich protein 1 isoform X3
aspartate-rich protein 1 isoform X4
aspartate-rich protein 1 isoform X5
aspartate-rich protein 1 isoform X6
ataxin-1 Alt-ATXN1
atherin-like
basic proline-rich protein
basic proline-rich protein-like
basic proline-rich protein-like
basic proline-rich protein-like
basic proline-rich protein-like
basic proline-rich protein-like
basic salivary proline-rich protein 1 isoform 1 preproprotein
basic salivary proline-rich protein 1 isoform 2 precursor
basic salivary proline-rich protein 1 isoform 3 precursor
basic salivary proline-rich protein 2 preproprotein
basic salivary proline-rich protein 3-like isoform X2
basic salivary proline-rich protein 3-like isoform X4
basic salivary proline-rich protein 3 precursor
basic salivary proline-rich protein 4 isoform 1 preproprotein
basic salivary proline-rich protein 4 isoform 2 precursor
basic salivary proline-rich protein 4-like isoform X3
bcl-2-binding component 3 isoform 1
beckwith-Wiedemann syndrome chromosomal region 1 candidate gene B protein isoform a
beckwith-Wiedemann syndrome chromosomal region 1 candidate gene B protein isoform b
beckwith-Wiedemann syndrome chromosomal region 1 candidate gene B protein isoform X1
beckwith-Wiedemann syndrome chromosomal region 1 candidate gene B protein isoform X2

beckwith-Wiedemann syndrome chromosomal region 1 candidate gene B protein isoform X3
beckwith-Wiedemann syndrome chromosomal region 1 candidate gene B protein isoform X3
beckwith-Wiedemann syndrome chromosomal region 1 candidate gene B protein isoform X3
BTB/POZ domain-containing protein 19 isoform X1
BTB/POZ domain-containing protein 19 isoform X6
BTB/POZ domain-containing protein KCTD15 isoform X2
cadmium/zinc-transporting ATPase HMA2-like
cancer/testis antigen 2 isoform LAGE-1b
casein kinase II subunit alpha'-interacting protein
CASP-like protein 4A1 isoform X1
cementoblastoma-derived protein 1
charged multivesicular body protein 1a isoform 1
cilia- and flagella-associated protein 61 isoform X15
cilia- and flagella-associated protein 61 isoform X16
cilia- and flagella-associated protein 61 isoform X17
cilia- and flagella-associated protein 61 isoform X18
cilia- and flagella-associated protein 61 isoform X19
CMT1A duplicated region transcript 15 protein isoform 1
cob(I)yrinic acid a,c-diamide adenosyltransferase, mitochondrial isoform X2
coiled-coil domain-containing protein 140
coiled-coil domain-containing protein 144B isoform X3
coiled-coil domain-containing protein 144B isoform X3
coiled-coil domain-containing protein 144B isoform X3
coiled-coil domain-containing protein 144B isoform X3
coiled-coil domain-containing protein 86-like
collagen alpha-1(I) chain
collagen alpha-1(I) chain-like
collagen alpha-1(III) chain-like
collagen alpha-1(III) chain-like
collagen alpha-1(III) chain-like
collagen alpha-1(VII) chain-like isoform X1
collagen alpha-2(I) chain-like
collagen alpha-2(IV) chain-like
collagen alpha-3(IX) chain-like
collagen alpha-3(IX) chain-like
collagen alpha-3(IX) chain-like
collagen alpha-3(IX) chain-like
collagen alpha-3(IX) chain-like
collagen alpha-3(IX) chain-like
cryptic family protein 1B isoform X1
C-type lectin domain family 17, member A isoform X5
C-type lectin domain family 17, member A isoform X6
cyclic AMP-responsive element-binding protein 5 isoform X7
cysteine-rich PAK1 inhibitor
cytokine-like protein 1 isoform X1

D-amino acid oxidase activator isoform 1
D-beta-hydroxybutyrate dehydrogenase, mitochondrial isoform X5
DDB1- and CUL4-associated factor 16
DDB1- and CUL4-associated factor 16
DDB1- and CUL4-associated factor 16
DDB1- and CUL4-associated factor 16
DDB1- and CUL4-associated factor 16
DDB1- and CUL4-associated factor 16
DDB1- and CUL4-associated factor 16 isoform X1
dendritic cell nuclear protein 1
dexamethasone-induced protein isoform X1
DNA-directed RNA polymerase II subunit RPB1-like
DNA-directed RNA polymerase II subunit RPB1-like
DNA transposase THAP9 isoform 2
DNA transposase THAP9 isoform X1
DNA transposase THAP9 isoform X2
DNA transposase THAP9 isoform X2
doublecortin domain-containing protein 1 isoform X11
doublecortin domain-containing protein 1 isoform X12
doublecortin domain-containing protein 1 isoform X13
dynactin-associated protein isoform 1
dynactin-associated protein isoform X2
E3 ubiquitin-protein ligase MARCH2 isoform X4
E3 ubiquitin-protein ligase MARCH3 isoform X4
elastin isoform b precursor
elastin isoform c precursor
elastin isoform d precursor
elastin isoform h precursor
elastin isoform k precursor
elastin isoform X11
elastin isoform X12
elastin isoform X14
elastin isoform X1
elastin isoform X2
elastin isoform X3
elastin isoform X4
elastin isoform X5
elastin isoform X6
elastin isoform X7
elastin isoform X9
elongation of very long chain fatty acids protein 7 isoform X3
elongation of very long chain fatty acids protein 7 isoform X4
endogenous Bornavirus-like nucleoprotein 1
endogenous retrovirus group K member 19 Env polyprotein-like
endogenous retrovirus group K member 6 Env polyprotein

endogenous retrovirus group K member 8 Gag polyprotein-like
endonuclease V isoform X20
endonuclease V isoform X20
endonuclease V isoform X20
endonuclease V isoform X20
enolase-phosphatase E1
envoplakin-like protein isoform X4
eukaryotic translation initiation factor 5A-1 isoform X2
eukaryotic translation initiation factor 5A-1 isoform X2
eukaryotic translation initiation factor 5A-1 isoform X2
eukaryotic translation initiation factor 5A-1 isoform X2
eukaryotic translation initiation factor 5A-1 isoform X2
exonuclease mut-7 homolog isoform b
FAM231A/C-like protein LOC102723383
FAM231A/C-like protein LOC102723383
Fanconi anemia core complex-associated protein 20 isoform 1
Fanconi anemia core complex-associated protein 20 isoform 3
fetal and adult testis-expressed transcript protein
flocculation protein FLO11-like
formiminotransferase N-terminal subdomain-containing protein isoform X1
formiminotransferase N-terminal subdomain-containing protein isoform X1
formiminotransferase N-terminal subdomain-containing protein isoform X1
formin-2-like
formin-2-like
formin-2-like
FOXL2 neighbor protein
FOXL2 neighbor protein isoform X1
FOXL2 neighbor protein isoform X1
FOXL2 neighbor protein isoform X2
glucose-dependent insulinotropic receptor-like
glutamate-rich protein 1 isoform X21
glutamate-rich protein 1 isoform X21
glutamate-rich protein 1 isoform X21
glutamate-rich protein 1 isoform X21
glutamate-rich protein 1 isoform X21
glutamate-rich protein 3 isoform X3
glutamate-rich protein 3 isoform X4
glutamate-rich protein 3 isoform X5
glutamate-rich protein 3 isoform X5
glutamate-rich protein 3 isoform X5
glutamate-rich protein 3 isoform X5
glutamate-rich protein 3 isoform X5
glutamate-rich protein 3 isoform X5
glutamate-rich protein 3 isoform X5
glutamate-rich protein 3 isoform X5

glycodelin isoform 2 precursor
glycodelin isoform X2
glycodelin isoform X4
glycoprotein Xg isoform 1 precursor
glycoprotein Xg isoform 2 precursor
glycoprotein Xg isoform 3 precursor
glycoprotein Xg isoform X1
glycoprotein Xg isoform X2
golgin subfamily A member 6-like protein 9 isoform 2
grainyhead-like protein 2 homolog isoform X3
granulysin isoform 1 precursor
granulysin isoform X1
granulysin isoform X2
granulysin isoform X3
H19 opposite tumor suppressor
helicase SRCAP-like
helix-loop-helix protein 2 isoform X1
heme transporter HRG1 isoform X1
heme transporter HRG1 isoform X1
hepatitis A virus cellular receptor 1 isoform X4
HERV-H LTR-associating protein 3 isoform X1
histidine-rich protein PFHRP-II-like
histone-lysine N-methyltransferase SETMAR isoform 5
histone-lysine N-methyltransferase SETMAR isoform 6
histone-lysine N-methyltransferase SETMAR isoform X1
histone-lysine N-methyltransferase SETMAR isoform X2
homeobox protein Hox-D13 isoform X2
hydroxyacyl-thioester dehydratase type 2, mitochondrial
hydroxyacyl-thioester dehydratase type 2, mitochondrial
hydroxyacyl-thioester dehydratase type 2, mitochondrial
hydroxyacyl-thioester dehydratase type 2, mitochondrial
hydroxysteroid 11-beta-dehydrogenase 1-like protein isoform h precursor
interleukin-26 precursor
interleukin-32 isoform B
interleukin-32 isoform B
interleukin-32 isoform B
interleukin-32 isoform B
interleukin-32 isoform C
interleukin-32 isoform C
interleukin-32 isoform D
interleukin-32 isoform E precursor
interleukin-3 precursor
keratin-associated protein 5-1
KRAB-A domain-containing protein 2 isoform X2
KRAB-A domain-containing protein 2 isoform X2

KRAB-A domain-containing protein 2 isoform X2
KRAB-A domain-containing protein 2 isoform X3
laforin-like
leucine-rich repeat-containing protein 53 isoform X3
leucine zipper protein 4 isoform 1
leucine zipper protein 4 isoform 2
leukocyte surface antigen CD47 isoform X3
lipocalin-like 1 protein
loricrin
loricrin isoform X1
LOW QUALITY PROTEIN: nascent polypeptide-associated complex subunit alpha, muscle-specific form-like
lymphocyte antigen 6K isoform X1
lymphocyte antigen 6K isoform X2
lymphocyte function-associated antigen 3 isoform 1
lymphocyte function-associated antigen 3 isoform 2
lymphocyte function-associated antigen 3 isoform X1
MAGE-like protein 2 isoform X2
MAGE-like protein 2 isoform X2
MAGE-like protein 2 isoform X3
MAGE-like protein 2 isoform X4
MAPK-interacting and spindle-stabilizing protein-like
melanoma-associated antigen C1-like
melanoma-associated antigen C1-like
melanoma-associated antigen C1-like
melanoma-associated antigen C1-like
membrane progestin receptor delta isoform 4
membrane progestin receptor delta isoform 7
membrane progestin receptor delta isoform 7
membrane progestin receptor delta isoform 7
membrane progestin receptor delta isoform 7
membrane progestin receptor delta isoform 7
membrane progestin receptor delta isoform X5
membrane progestin receptor delta isoform X5
membrane progestin receptor delta isoform X5
membrane progestin receptor delta isoform X5
methyl-CpG-binding domain protein 6-like, partial
mitochondrial basic amino acids transporter isoform X5
mitochondrial enolase superfamily member 1 isoform 3
mitochondrial enolase superfamily member 1 isoform 4
mitochondrial enolase superfamily member 1 isoform 5
mitochondrial enolase superfamily member 1 isoform 6
mitochondrial enolase superfamily member 1 isoform 7
mitochondrial enolase superfamily member 1 isoform rTSalpha
mitochondrial enolase superfamily member 1 isoform rTSbeta
mitochondrial enolase superfamily member 1 isoform X10

mitochondrial enolase superfamily member 1 isoform X11
mitochondrial enolase superfamily member 1 isoform X12
mitochondrial enolase superfamily member 1 isoform X13
mitochondrial enolase superfamily member 1 isoform X13
mitochondrial enolase superfamily member 1 isoform X14
mitochondrial enolase superfamily member 1 isoform X15
mitochondrial enolase superfamily member 1 isoform X16
mitochondrial enolase superfamily member 1 isoform X17
mitochondrial enolase superfamily member 1 isoform X18
mitochondrial enolase superfamily member 1 isoform X18
mitochondrial enolase superfamily member 1 isoform X18
mitochondrial enolase superfamily member 1 isoform X19
mitochondrial enolase superfamily member 1 isoform X1
mitochondrial enolase superfamily member 1 isoform X2
mitochondrial enolase superfamily member 1 isoform X3
mitochondrial enolase superfamily member 1 isoform X4
mitochondrial enolase superfamily member 1 isoform X5
mitochondrial enolase superfamily member 1 isoform X6
mitochondrial enolase superfamily member 1 isoform X7
mitochondrial enolase superfamily member 1 isoform X8
mitochondrial enolase superfamily member 1 isoform X9
mitogen-activated protein kinase 7-like
mucin-1 isoform 8 precursor
mucin-1-like
mucin-1-like
mucin-1-like
mucin-5AC-like isoform X2
mucin-7 precursor
mucin-7 precursor
mucin-7 precursor
myeloma-overexpressed gene protein isoform 1
myeloma-overexpressed gene protein isoform 1
myeloma-overexpressed gene protein isoform 2
myeloma-overexpressed gene protein isoform 2
myeloma-overexpressed gene protein isoform 2
myeloma-overexpressed gene protein isoform 2
myoD family inhibitor domain-containing protein isoform c
myosin heavy chain IB-like
NANOG neighbor homeobox
nascent polypeptide-associated complex subunit alpha, muscle-specific form-like
natural cytotoxicity triggering receptor 3 isoform a precursor
natural cytotoxicity triggering receptor 3 isoform b precursor
natural cytotoxicity triggering receptor 3 isoform c precursor
natural cytotoxicity triggering receptor 3 isoform X1
natural cytotoxicity triggering receptor 3 isoform X2

natural cytotoxicity triggering receptor 3 ligand 1-like isoform X1
N-cym protein isoform X1
netrin-G2 isoform X7
neuroblastoma breakpoint family member 20
neurocalcin-delta isoform X1
non-histone chromosomal protein HMG-14 isoform X1
non-histone chromosomal protein HMG-14 isoform X1
non-histone chromosomal protein HMG-14 isoform X1
non-histone chromosomal protein HMG-14 isoform X1
non-histone chromosomal protein HMG-14 isoform X1
non-histone chromosomal protein HMG-14 isoform X1
non-histone chromosomal protein HMG-14 isoform X1
non-histone chromosomal protein HMG-14 isoform X1
non-histone chromosomal protein HMG-14 isoform X1
non-histone chromosomal protein HMG-14 isoform X1
non-histone chromosomal protein HMG-14 isoform X1
non-histone chromosomal protein HMG-14 isoform X1
nuclear pore complex-interacting protein family member A1
nuclear pore complex-interacting protein family member A1-like isoform X3
nuclear pore complex-interacting protein family member A1-like isoform X4
nuclear pore complex-interacting protein family member A1-like isoform X5
nuclear pore complex-interacting protein family member A1-like isoform X7
nuclear pore complex-interacting protein family member A2
nuclear pore complex-interacting protein family member A2 isoform X10
nuclear pore complex-interacting protein family member A2 isoform X2
nuclear pore complex-interacting protein family member A2 isoform X5
nuclear pore complex-interacting protein family member A2 isoform X9
nuclear pore complex-interacting protein family member A3
nuclear pore complex-interacting protein family member A3 isoform X2
nuclear pore complex-interacting protein family member A3 isoform X4
nuclear pore complex-interacting protein family member A3 isoform X8
nuclear pore complex-interacting protein family member A3 isoform X9
nuclear pore complex-interacting protein family member A5 isoform 1
nuclear pore complex-interacting protein family member A5 isoform X4
nuclear pore complex-interacting protein family member A5-like isoform X8
nuclear pore complex-interacting protein family member A7 isoform X1
nuclear pore complex-interacting protein family member A7 isoform X2
nuclear pore complex-interacting protein family member A7 isoform X3
nuclear pore complex-interacting protein family member A7 isoform X4
nuclear pore complex-interacting protein family member A7 isoform X4
nuclear pore complex-interacting protein family member A7 isoform X5
nuclear pore complex-interacting protein family member A7 isoform X6
nuclear pore complex-interacting protein family member A7 isoform X6
nuclear pore complex-interacting protein family member A7 isoform X7
nuclear pore complex-interacting protein family member A7 isoform X8

nuclear pore complex-interacting protein family member A7 isoform X9
nuclear pore complex-interacting protein family member A7-like isoform X1
nuclear pore complex-interacting protein family member A7-like isoform X2
nuclear pore complex-interacting protein family member A7-like isoform X6
nuclear pore complex-interacting protein family member A8
nuclear pore complex-interacting protein family member A8
nuclear pore complex-interacting protein family member B11 isoform X3
nuclear pore complex-interacting protein family member B15 isoform X1
nuclear pore complex-interacting protein family member B15 isoform X2
nuclear pore complex-interacting protein family member B15 isoform X2
nuclear pore complex-interacting protein family member B15 isoform X3
nuclear pore complex-interacting protein family member B15 isoform X4
nuclear pore complex-interacting protein family member B15 precursor
nuclear pore complex-interacting protein family member B4 isoform X3
nuclear pore complex-interacting protein family member B4 isoform X6
nuclear pore complex-interacting protein family member B4 isoform X7
nuclear pore complex-interacting protein family member B4 isoform X7
nuclear pore complex-interacting protein family member B6 isoform X2
nuclear pore complex-interacting protein family member B6 isoform X3
nuclear pore complex-interacting protein family member B6 isoform X3
nuclear pore complex-interacting protein family member B6 isoform X3
nuclear pore complex-interacting protein family member B6 isoform X4
nuclear pore complex-interacting protein family member B6 isoform X5
nuclear pore complex-interacting protein family member B6 isoform X7
nuclear pore complex-interacting protein family member B8 isoform X4
nuclear pore complex-interacting protein family member B8 isoform X5
nuclear pore complex-interacting protein family member B8 isoform X5
nuclear pore complex-interacting protein family member B8 isoform X6
nuclear pore complex-interacting protein family member B8 isoform X7
nuclear pore complex-interacting protein family member B9 isoform 2
nuclear pore complex-interacting protein family member B9 isoform X11
nuclear pore complex-interacting protein family member B9 isoform X12
nuclear pore complex-interacting protein family member B9 isoform X13
nuclear pore complex-interacting protein family member B9 isoform X14
nuclear pore complex-interacting protein family member B9 isoform X5
nuclear pore complex-interacting protein family member B9 isoform X6
nuclear pore complex-interacting protein family member B9 isoform X7
nuclear pore complex-interacting protein family member B9 isoform X7
nuclear pore complex-interacting protein family member B9 isoform X7
nuclear pore complex-interacting protein family member B9 isoform X9
nuclear receptor subfamily 4 group A member 1 isoform X4
nuclear receptor subfamily 4 group A member 1 isoform X5
odontogenesis associated phosphoprotein isoform 1 precursor
odontogenesis associated phosphoprotein isoform X1
opiorphin prepropeptide isoform 1 precursor

paraneoplastic antigen Ma6E-like
phosphatidylethanolamine-binding protein 4 isoform X2
phosphatidylinositol 4,5-bisphosphate 5-phosphatase A-like
phospholipase A2 inhibitor and Ly6/PLAUR domain-containing protein-like
phospholipase A2 inhibitor and Ly6/PLAUR domain-containing protein-like
phospholipase A2 inhibitor and Ly6/PLAUR domain-containing protein-like
phospholipase A2 inhibitor and Ly6/PLAUR domain-containing protein-like
phospholipase A2 inhibitor and Ly6/PLAUR domain-containing protein-like
phospholipase A2 inhibitor and Ly6/PLAUR domain-containing protein-like
platelet-activating factor acetylhydrolase IB subunit alpha isoform X4
post-GPI attachment to proteins factor 2 isoform 10
post-GPI attachment to proteins factor 2 isoform 13
post-GPI attachment to proteins factor 2 isoform 14
post-GPI attachment to proteins factor 2 isoform X16
post-GPI attachment to proteins factor 2 isoform X17
post-GPI attachment to proteins factor 2 isoform X20
post-GPI attachment to proteins factor 2 isoform X20
post-GPI attachment to proteins factor 2 isoform X20
post-GPI attachment to proteins factor 2 isoform X21
pre T-cell antigen receptor alpha isoform 4 precursor
pre T-cell antigen receptor alpha isoform X3
pre T-cell antigen receptor alpha isoform X4
pre T-cell antigen receptor alpha isoform X5
proapoptotic nucleolar protein 1
probable gluconokinase isoform X5
probable N-acetyltransferase 16
probable N-acetyltransferase 16 isoform X1
probable N-acetyltransferase 16 isoform X2
probable palmitoyltransferase ZDHHC11 isoform X14
probable palmitoyltransferase ZDHHC11 isoform X15
probable serine/threonine-protein kinase DDB_G0277071 isoform X2
probable serine/threonine-protein kinase DDB_G0277071 isoform X3
probable serine/threonine-protein kinase DDB_G0277071 isoform X3
probable serine/threonine-protein kinase DDB_G0277071 isoform X4
proline-rich extensin-like protein EPR1
proline-rich extensin-like protein EPR1
proline-rich protein 18-like
proline-rich protein 20A
proline-rich protein 20B
proline-rich protein 20C
proline-rich protein 20D
proline-rich protein 20E
proline-rich protein 23D1
proline-rich protein 23D1 isoform X1
proline-rich protein 23D2

proline-rich protein 23D2 isoform X1
proline-rich protein 25
proline-rich protein 26 isoform 1
proline-rich protein 26 isoform 2
proline-rich protein 26 isoform 3
proline-rich protein 26 isoform X1
proline-rich protein 26 isoform X2
proline-rich protein 26 isoform X3
proline-rich protein 31
proline-rich protein 36-like isoform X1
proline-rich protein 36-like isoform X1
proline-rich protein, Y-linked
proline-rich protein, Y-linked isoform X1
proline-rich protein, Y-linked isoform X1
proline-rich proteoglycan 2 isoform X4
proline-rich proteoglycan 2-like
proline-rich proteoglycan 2-like
protein app1-like
protein app1-like
protein CEI isoform X1
protein CROC-4 isoform 1
protein CROC-4 isoform X1
protein CROC-4 isoform X1
protein CROC-4 isoform X2
protein CROC-4 isoform X3
protein CROC-4 isoform X3
protein CROC-4 isoform X3
protein CROC-4 isoform X3
protein CROC-4 isoform X3
protein enabled homolog
protein enabled homolog
protein FAM106A
protein FAM153A
protein FAM153A isoform X1
protein FAM153A isoform X1
protein FAM153A isoform X1
protein FAM153A isoform X1
protein FAM153A isoform X1
protein FAM153A isoform X1
protein FAM153A isoform X1
protein FAM153A isoform X1
protein FAM153A isoform X1
protein FAM153A isoform X1
protein FAM153A isoform X1
protein FAM153A isoform X2

protein FAM153A isoform X3
protein FAM153A isoform X4
protein FAM153A isoform X4
protein FAM153A isoform X5
protein FAM153A isoform X6
protein FAM153A isoform X6
protein FAM153A isoform X6
protein FAM153A isoform X6
protein FAM153A isoform X6
protein FAM153A isoform X6
protein FAM153A isoform X6
protein FAM153A isoform X6
protein FAM153A isoform X6
protein FAM153A isoform X7
protein FAM153B
protein FAM218A
protein FAM231C
protein FAM231D
protein FAM231D
protein FAM231D
protein FAM231D
protein FAM32A isoform X1
protein GDF5OS, mitochondrial precursor
protein SPT2 homolog
protein ZNF365 isoform D
protein ZNF365 isoform X2
protein ZNF365 isoform X3
protein ZNF365 isoform X4
protein ZNF365 isoform X5
psoriasis susceptibility 1 candidate gene 1 protein
putative bifunctional UDP-N-acetylglucosamine transferase and deubiquitinase ALG13 isoform X15
putative bifunctional UDP-N-acetylglucosamine transferase and deubiquitinase ALG13 isoform X16
putative coiled-coil domain-containing protein 144 N-terminal-like
putative coiled-coil domain-containing protein 144 N-terminal-like isoform X1
putative coiled-coil domain-containing protein 144 N-terminal-like isoform X2
putative coiled-coil domain-containing protein 144 N-terminal-like isoform X3
putative cuticle collagen 91
putative golgin subfamily A member 8I
putative golgin subfamily A member 8I
putative golgin subfamily A member 8I
putative golgin subfamily A member 8I
putative golgin subfamily A member 8I
putative hydroxypyruvate isomerase isoform 1
putative hydroxypyruvate isomerase isoform 3
putative hydroxypyruvate isomerase isoform 4

putative hydroxypyruvate isomerase isoform 5
putative hydroxypyruvate isomerase isoform X1
putative hydroxypyruvate isomerase isoform X2
putative hydroxypyruvate isomerase isoform X3
putative inactive deoxyuridine 5'-triphosphate nucleotidohydrolase-like protein FLJ16323 isoform X1, partial
putative NPIP-like protein LOC729978
putative NPIP-like protein LOC729978 isoform X1
putative NPIP-like protein LOC729978 isoform X1
putative NPIP-like protein LOC729978 isoform X1
putative NPIP-like protein LOC729978 isoform X2
putative NPIP-like protein LOC729978 isoform X3
putative NPIP-like protein LOC729978 isoform X3
putative NPIP-like protein LOC729978 isoform X4
putative NPIP-like protein LOC729978 isoform X5
putative POM121-like protein 1-like
putative POM121-like protein 1-like
putative POM121-like protein 1-like
putative POM121-like protein 1-like
putative POM121-like protein 1-like
putative POM121-like protein 1-like
putative POM121-like protein 1-like
putative POM121-like protein 1-like
putative POM121-like protein 1-like
putative POM121-like protein 1-like
putative protein FAM106C
putative protein FAM157B isoform X1
putative protein FAM231BP
putative TBC1 domain family member 29
putative TBC1 domain family member 29 isoform X1
putative uncharacterized protein C10orf113 isoform 1
putative uncharacterized protein C10orf126
putative uncharacterized protein C11orf40
putative uncharacterized protein C12orf77 isoform X1
putative uncharacterized protein C15orf56
putative uncharacterized protein C17orf82
putative uncharacterized protein C18orf65
putative uncharacterized protein C1orf229
putative uncharacterized protein C20orf78 isoform a
putative uncharacterized protein C22orf34
putative uncharacterized protein C3orf56
putative uncharacterized protein C6orf99 isoform X1
putative uncharacterized protein C6orf99 isoform X2
putative uncharacterized protein C7orf71 isoform a
putative uncharacterized protein C7orf71 isoform X1
putative uncharacterized protein C7orf71 isoform X1

putative uncharacterized protein C7orf71 isoform X2
putative uncharacterized protein C8orf44
putative uncharacterized protein C8orf49
putative uncharacterized protein C9orf62
putative uncharacterized protein CCDC197 isoform X1
putative uncharacterized protein CCDC197 isoform X1
putative uncharacterized protein encoded by LINC00205
putative uncharacterized protein encoded by LINC00483 isoform X1
putative uncharacterized protein encoded by LINC00483 isoform X1
putative uncharacterized protein encoded by LINC00483 isoform X1
putative uncharacterized protein encoded by LINC00483 isoform X1
putative uncharacterized protein FLJ35723 isoform a
putative uncharacterized protein FLJ35723 isoform b
putative uncharacterized protein FLJ35723 isoform X1
putative uncharacterized protein FLJ35723 isoform X2
putative uncharacterized protein FLJ44672
putative uncharacterized protein FLJ44672
putative uncharacterized protein FLJ44672
putative uncharacterized protein FLJ44672
putative uncharacterized protein FLJ44672
putative uncharacterized protein FLJ44672
putative uncharacterized protein FLJ44672
putative uncharacterized protein FLJ44672
putative uncharacterized protein FLJ44672
putative uncharacterized protein FLJ44672
putative uncharacterized protein FLJ44672
putative uncharacterized protein FLJ44672
putative uncharacterized protein FLJ44672
putative uncharacterized protein FLJ44672 isoform X2
putative uncharacterized protein FLJ44672 isoform X2
putative uncharacterized protein FLJ44672 isoform X3
putative uncharacterized protein FLJ44672, partial
putative uncharacterized protein FLJ44790
putative uncharacterized protein FLJ46204
putative uncharacterized protein FLJ46235
putative uncharacterized protein FLJ92257
putative uncharacterized protein FLJ92257
putative uncharacterized protein LOC388882
putative uncharacterized protein LOC388882
putative uncharacterized protein LOC388882
putative uncharacterized protein LOC388882
putative uncharacterized protein LOC388882
putative uncharacterized protein LOC388882
putative uncharacterized protein LOC388882
putative uncharacterized protein LOC388882

putative uncharacterized protein LOC388882
putative uncharacterized protein LOC388882
putative uncharacterized protein LOC388882
putative uncharacterized protein LOC388882
putative uncharacterized protein LOC388882
putative uncharacterized protein LOC388882
putative uncharacterized protein LOC388882
putative uncharacterized protein LOC388882
putative uncharacterized protein LOC400499 isoform X13
putative uncharacterized protein LOC400499 isoform X15
putative uncharacterized protein LOC400499 isoform X18
putative uncharacterized protein LOC400499 isoform X19
putative uncharacterized protein LOC439951
putative uncharacterized protein MGC34800
putative uncharacterized protein MGC39545
putative uncharacterized protein UNQ6494/PRO21346
putative uncharacterized protein UNQ9370/PRO34162
putative UPF0607 protein ENSP00000383144
putative UPF0607 protein ENSP00000383783
putative UPF0607 protein FLJ37424
ras-related protein Rab-35 isoform X1
ras-related protein Rab-35 isoform X1
reelin domain-containing protein 1 isoform X2
regulator of hemoglobinization and erythroid cell expansion protein
regulator of hemoglobinization and erythroid cell expansion protein isoform X1
rho guanine nucleotide exchange factor 4 isoform X4
rhox homeobox family member 2 isoform X1
RING finger protein 166 isoform X1
RNA-binding protein 24 isoform X2
RNA-binding protein 3 isoform X1
RNA-binding protein 3 isoform X1
salivary acidic proline-rich phosphoprotein 1/2 isoform a preproprotein
salivary acidic proline-rich phosphoprotein 1/2 isoform b
salivary acidic proline-rich phosphoprotein 1/2 preproprotein
serine/arginine repetitive matrix protein 1
serine/arginine repetitive matrix protein 1-like
serine/arginine repetitive matrix protein 2-like isoform X7
serine/arginine-rich splicing factor RSZ22-like
serine/arginine-rich splicing factor RSZ22-like isoform X8
serine palmitoyltransferase small subunit B isoform X1
serine palmitoyltransferase small subunit B isoform X1
serine protease inhibitor Kazal-type 2 isoform X1
serine/threonine-protein kinase 32C isoform X4
SET domain-containing protein 9 isoform 1
SET domain-containing protein 9 isoform 2

SET domain-containing protein 9 isoform 3
SET domain-containing protein 9 isoform 4
SET domain-containing protein 9 isoform 5
SET domain-containing protein 9 isoform X1
SET domain-containing protein 9 isoform X2
SET domain-containing protein 9 isoform X3
sine oculis-binding protein homolog
single-pass membrane and coiled-coil domain-containing protein 4 isoform X1
single-pass membrane and coiled-coil domain-containing protein 4 isoform X2
single-pass membrane and coiled-coil domain-containing protein 4 isoform X3
single-pass membrane and coiled-coil domain-containing protein 4 isoform X4
single-pass membrane and coiled-coil domain-containing protein 4 isoform X5
single-pass membrane and coiled-coil domain-containing protein 4 isoform X5
small integral membrane protein 27 isoform X1
small integral membrane protein 28
small integral membrane protein 5 isoform X1
small integral membrane protein 5 isoform X2
sperm acrosome-associated protein 7 isoform X7
sperm acrosome-associated protein 7 isoform X8
spermatogenesis-associated protein 12
spermatogenesis-associated protein 12 isoform X1
sperm protein associated with the nucleus on the X chromosome N4 isoform X1
sperm protein associated with the nucleus on the X chromosome N4 isoform X2
synapsin-1-like
synapsin-1-like
TBC1 domain family member 28 isoform X4
T-cell acute lymphocytic leukemia protein 1 isoform X2
TCR gamma alternate reading frame protein isoform X1
tetraspanin-32 isoform X12
THAP domain-containing protein 5 isoform 3
THAP domain-containing protein 5 isoform 3
THAP domain-containing protein 5 isoform 3
TP53 target 3 family member E isoform X1
TP53 target 3 family member E isoform X1
TP53 target 3 family member F isoform X1
TP53 target 3 family member F isoform X1
TP53-target gene 3 protein isoform X1
TP53-target gene 3 protein isoform X1
TP53-target gene 3 protein isoform X1
TP53-target gene 3 protein isoform X1
TP53-target gene 3 protein isoform X1
TP53-target gene 3 protein isoform X1
TP53-target gene 3 protein isoform X1
transcription factor NF-E4
translation initiation factor IF-2-like

translation initiation factor IF-2-like
translation initiation factor IF-2-like
translation initiation factor IF-2-like
translation initiation factor IF-2-like isoform X1
translation initiation factor IF-2-like isoform X1
transmembrane and immunoglobulin domain-containing protein 2 isoform 1 precursor
transmembrane and immunoglobulin domain-containing protein 2 isoform 2 precursor
transmembrane and immunoglobulin domain-containing protein 2 isoform 3
transmembrane and immunoglobulin domain-containing protein 2 isoform X1
transmembrane and immunoglobulin domain-containing protein 2 isoform X2
transmembrane protein 108-like
transmembrane protein 127 isoform X1
transmembrane protein 184A isoform X1
transmembrane protein 187
transmembrane protein 187 isoform X1
transmembrane protein 191A
transmembrane protein 205 isoform X1
transmembrane protein 225B
transmembrane protein 225B
transmembrane protein 225B
transmembrane protein 225B isoform X1
transmembrane protein 225B isoform X2
transmembrane protein 225B isoform X2
transmembrane protein 225B isoform X2
transmembrane protein 225B isoform X2
transmembrane protein 225B isoform X2
transmembrane protein 225B isoform X2
transmembrane protein 225B isoform X2
transmembrane protein 225B isoform X2
transmembrane protein 225B isoform X3
transmembrane protein 225B isoform X3
transmembrane protein 225B isoform X3
transmembrane protein 225B isoform X3
transmembrane protein 225B isoform X3
transmembrane protein 225B isoform X3
transmembrane protein 241 isoform X6
transmembrane protein 272 isoform 1
transmembrane protein 31
transmembrane protein 99 isoform X1
transmembrane protein 99 isoform X1
transmembrane protein 99 precursor
transmembrane protein C17orf113
transmembrane protein CCDC163 isoform 3
TRIO and F-actin-binding protein-like
TRIO and F-actin-binding protein-like

TRIO and F-actin-binding protein-like
tRNA (adenine(58)-N(1))-methyltransferase, mitochondrial isoform X2
tRNA (adenine(58)-N(1))-methyltransferase, mitochondrial isoform X3
tRNA (adenine(58)-N(1))-methyltransferase, mitochondrial isoform X4
tRNA (adenine(58)-N(1))-methyltransferase, mitochondrial isoform X5
tudor domain-containing protein 10 isoform a
tudor domain-containing protein 10 isoform b
tudor domain-containing protein 10 isoform X10
tudor domain-containing protein 10 isoform X1
tudor domain-containing protein 10 isoform X1
tudor domain-containing protein 10 isoform X2
tudor domain-containing protein 10 isoform X3
tudor domain-containing protein 10 isoform X4
tudor domain-containing protein 10 isoform X5
tudor domain-containing protein 10 isoform X6
tudor domain-containing protein 10 isoform X6
tudor domain-containing protein 10 isoform X7
tudor domain-containing protein 10 isoform X8
tudor domain-containing protein 10 isoform X9
tudor domain-containing protein 10 isoform X9
tudor domain-containing protein 10 isoform X9
tumor necrosis factor alpha-induced protein 2 isoform X3
tumor necrosis factor receptor superfamily member 14 isoform X1
tumor necrosis factor receptor superfamily member 14 isoform X2
type-1 angiotensin II receptor-associated protein isoform d
type-1 angiotensin II receptor-associated protein isoform X2
ubiquitin-conjugating enzyme E2 E2 isoform X3
ubiquitin-conjugating enzyme E2 E2 isoform X4
ubiquitin-conjugating enzyme E2 E2 isoform X5
UDP-GalNAc:beta-1,3-N-acetylgalactosaminyltransferase 1 isoform X2
uncharacterized protein C10orf111
uncharacterized protein C10orf55
uncharacterized protein C10orf68 isoform X28
uncharacterized protein C10orf68 isoform X28
uncharacterized protein C10orf68 isoform X28
uncharacterized protein C10orf68 isoform X29
uncharacterized protein C10orf68 isoform X32
uncharacterized protein C10orf68 isoform X33
uncharacterized protein C10orf68 isoform X35
uncharacterized protein C10orf68 isoform X38
uncharacterized protein C10orf68 isoform X39
uncharacterized protein C10orf68 isoform X40
uncharacterized protein C10orf68 isoform X41
uncharacterized protein C10orf68 isoform X43
uncharacterized protein C10orf68 isoform X44

uncharacterized protein C10orf95
uncharacterized protein C11orf21 isoform X1
uncharacterized protein C12orf40 isoform 1
uncharacterized protein C12orf40 isoform 2
uncharacterized protein C12orf40 isoform X1
uncharacterized protein C12orf40 isoform X1
uncharacterized protein C12orf74 isoform 1
uncharacterized protein C12orf74 isoform 2
uncharacterized protein C15orf32 isoform a
uncharacterized protein C15orf32 isoform X1
uncharacterized protein C15orf53
uncharacterized protein C16orf71
uncharacterized protein C16orf71 isoform X1
uncharacterized protein C16orf71 isoform X2
uncharacterized protein C16orf71 isoform X3
uncharacterized protein C17orf102
uncharacterized protein C17orf51
uncharacterized protein C17orf51 isoform X1
uncharacterized protein C17orf51 isoform X1
uncharacterized protein C17orf51 isoform X1
uncharacterized protein C17orf77
uncharacterized protein C17orf77
uncharacterized protein C18orf12
uncharacterized protein C20orf203
uncharacterized protein C20orf203 isoform X1
uncharacterized protein C21orf58 isoform X7
uncharacterized protein C22orf24 isoform a
uncharacterized protein C22orf24 isoform c
uncharacterized protein C22orf34 isoform X1
uncharacterized protein C22orf42
uncharacterized protein C22orf42 isoform X1
uncharacterized protein C22orf42 isoform X2
uncharacterized protein C22orf42 isoform X3
uncharacterized protein C22orf42 isoform X4
uncharacterized protein C22orf42 isoform X5
uncharacterized protein C2orf27A isoform 1
uncharacterized protein C2orf27A isoform 1
uncharacterized protein C2orf27B
uncharacterized protein C2orf48
uncharacterized protein C2orf91 isoform X1
uncharacterized protein C2orf92 isoform X1
uncharacterized protein C2orf92 isoform X2
uncharacterized protein C2orf92 isoform X3
uncharacterized protein C2orf92 isoform X4
uncharacterized protein C2orf92 isoform X5

uncharacterized protein C2orf92 isoform X6
uncharacterized protein C2orf92 isoform X7
uncharacterized protein C2orf92 precursor
uncharacterized protein C3orf18 isoform X3
uncharacterized protein C3orf18 isoform X3
uncharacterized protein C3orf18 isoform X3
uncharacterized protein C3orf18 isoform X3
uncharacterized protein C3orf18 isoform X3
uncharacterized protein C3orf18 isoform X3
uncharacterized protein C3orf18 isoform X3
uncharacterized protein C3orf36
uncharacterized protein C6orf223 isoform 1
uncharacterized protein C6orf223 isoform X1
uncharacterized protein C7orf33
uncharacterized protein C7orf65
uncharacterized protein C8orf86 isoform 1
uncharacterized protein C9orf139
uncharacterized protein C9orf139 isoform X1
uncharacterized protein C9orf163
uncharacterized protein C9orf47 isoform 1 precursor
uncharacterized protein C9orf47 isoform 2 precursor
uncharacterized protein C9orf57
uncharacterized protein C9orf66
uncharacterized protein CH507-42P11.6
uncharacterized protein CH507-42P11.6
uncharacterized protein CH507-42P11.6
uncharacterized protein CXorf67
uncharacterized protein ENSP00000382033
uncharacterized protein ENSP00000382042
uncharacterized protein ENSP00000382042-like
uncharacterized protein FAM120AOS isoform 1
uncharacterized protein FLJ20712
uncharacterized protein FLJ40521 isoform X1
uncharacterized protein FLJ40521 isoform X2
uncharacterized protein FLJ40521 isoform X3
uncharacterized protein FLJ40521-like
uncharacterized protein FLJ40521-like
uncharacterized protein FLJ46347-like
uncharacterized protein KIAA0040 isoform X1
uncharacterized protein KIAA0040 isoform X1
uncharacterized protein KIAA0040 isoform X1
uncharacterized protein KIAA0040 isoform X1
uncharacterized protein KIAA0040 isoform X1
uncharacterized protein KIAA0040 isoform X1
uncharacterized protein KIAA0040 isoform X1

uncharacterized protein KIAA1257 isoform a
uncharacterized protein KIAA1257 isoform b
uncharacterized protein KIAA1257 isoform c
uncharacterized protein KIAA1257 isoform d
uncharacterized protein KIAA1257 isoform d
uncharacterized protein KIAA1257 isoform X10
uncharacterized protein KIAA1257 isoform X1
uncharacterized protein KIAA1257 isoform X1
uncharacterized protein KIAA1257 isoform X1
uncharacterized protein KIAA1257 isoform X1
uncharacterized protein KIAA1257 isoform X2
uncharacterized protein KIAA1257 isoform X3
uncharacterized protein KIAA1257 isoform X4
uncharacterized protein KIAA1257 isoform X5
uncharacterized protein KIAA1257 isoform X6
uncharacterized protein KIAA1257 isoform X7
uncharacterized protein KIAA1257 isoform X7
uncharacterized protein KIAA1257 isoform X7
uncharacterized protein KIAA1257 isoform X8
uncharacterized protein KIAA1257 isoform X8
uncharacterized protein KIAA1257 isoform X8
uncharacterized protein KIAA1257 isoform X9
uncharacterized protein LOC100128124
uncharacterized protein LOC100129098
uncharacterized protein LOC100129697
uncharacterized protein LOC100129940 isoform 1
uncharacterized protein LOC100129940 isoform 2
uncharacterized protein LOC100130357
uncharacterized protein LOC100130370 isoform X1
uncharacterized protein LOC100130370 isoform X2
uncharacterized protein LOC100130370 isoform X3
uncharacterized protein LOC100130370 isoform X4
uncharacterized protein LOC100130449
uncharacterized protein LOC100131094
uncharacterized protein LOC100132874
uncharacterized protein LOC100133204
uncharacterized protein LOC100134391
uncharacterized protein LOC100134391 isoform X1
uncharacterized protein LOC100286986
uncharacterized protein LOC100287387
uncharacterized protein LOC100506571
uncharacterized protein LOC100507221
uncharacterized protein LOC100507507
uncharacterized protein LOC100652901
uncharacterized protein LOC100996259

uncharacterized protein LOC100996274
uncharacterized protein LOC100996318
uncharacterized protein LOC100996413
uncharacterized protein LOC100996413
uncharacterized protein LOC100996574
uncharacterized protein LOC100996598
uncharacterized protein LOC100996701
uncharacterized protein LOC100996720
uncharacterized protein LOC100996842 isoform 1
uncharacterized protein LOC100996842 isoform X1
uncharacterized protein LOC101060017
uncharacterized protein LOC101060017
uncharacterized protein LOC101060179
uncharacterized protein LOC101060341 isoform X1
uncharacterized protein LOC101060341 isoform X1
uncharacterized protein LOC101060588
uncharacterized protein LOC101060588
uncharacterized protein LOC101060588
uncharacterized protein LOC101927353
uncharacterized protein LOC101927353
uncharacterized protein LOC101927353
uncharacterized protein LOC101927353
uncharacterized protein LOC101927353
uncharacterized protein LOC101927353
uncharacterized protein LOC101927353
uncharacterized protein LOC101927401 isoform X1
uncharacterized protein LOC101927503 isoform X1
uncharacterized protein LOC101927503 isoform X1
uncharacterized protein LOC101927562
uncharacterized protein LOC101927824
uncharacterized protein LOC101927824
uncharacterized protein LOC101927824
uncharacterized protein LOC101928095 isoform X1
uncharacterized protein LOC101928095 isoform X1
uncharacterized protein LOC101928095 isoform X1
uncharacterized protein LOC101928095 isoform X1
uncharacterized protein LOC101928095 isoform X2
uncharacterized protein LOC101928095 isoform X3
uncharacterized protein LOC101928095 isoform X4
uncharacterized protein LOC101928095 isoform X5
uncharacterized protein LOC101928095 isoform X6
uncharacterized protein LOC101928120
uncharacterized protein LOC101928193 isoform X1
uncharacterized protein LOC101928193 isoform X2
uncharacterized protein LOC101928193 isoform X3

uncharacterized protein LOC101928212 isoform X1
uncharacterized protein LOC101928212 isoform X2
uncharacterized protein LOC101928268
uncharacterized protein LOC101928548
uncharacterized protein LOC101928589
uncharacterized protein LOC101928764 isoform X1
uncharacterized protein LOC101928764 isoform X2
uncharacterized protein LOC101929097
uncharacterized protein LOC101929372
uncharacterized protein LOC101929400 isoform X1
uncharacterized protein LOC101929400 isoform X2
uncharacterized protein LOC101929400 isoform X3
uncharacterized protein LOC101929692
uncharacterized protein LOC101929747
uncharacterized protein LOC101929895
uncharacterized protein LOC101929937
uncharacterized protein LOC101930307 isoform X10
uncharacterized protein LOC101930307 isoform X11
uncharacterized protein LOC101930307 isoform X12
uncharacterized protein LOC101930307 isoform X13
uncharacterized protein LOC101930307 isoform X14
uncharacterized protein LOC101930307 isoform X15
uncharacterized protein LOC101930307 isoform X16
uncharacterized protein LOC101930307 isoform X17
uncharacterized protein LOC101930307 isoform X18
uncharacterized protein LOC101930307 isoform X19
uncharacterized protein LOC101930307 isoform X1
uncharacterized protein LOC101930307 isoform X20
uncharacterized protein LOC101930307 isoform X21
uncharacterized protein LOC101930307 isoform X22
uncharacterized protein LOC101930307 isoform X23
uncharacterized protein LOC101930307 isoform X24
uncharacterized protein LOC101930307 isoform X25
uncharacterized protein LOC101930307 isoform X26
uncharacterized protein LOC101930307 isoform X26
uncharacterized protein LOC101930307 isoform X27
uncharacterized protein LOC101930307 isoform X28
uncharacterized protein LOC101930307 isoform X29
uncharacterized protein LOC101930307 isoform X2
uncharacterized protein LOC101930307 isoform X3
uncharacterized protein LOC101930307 isoform X4
uncharacterized protein LOC101930307 isoform X5
uncharacterized protein LOC101930307 isoform X6
uncharacterized protein LOC101930307 isoform X7
uncharacterized protein LOC101930307 isoform X8

uncharacterized protein LOC101930307 isoform X9
uncharacterized protein LOC102723360 isoform X1
uncharacterized protein LOC102723750 isoform X1
uncharacterized protein LOC102723750 isoform X1
uncharacterized protein LOC102723750 isoform X1
uncharacterized protein LOC102723750 isoform X1
uncharacterized protein LOC102723750 isoform X1
uncharacterized protein LOC102723750 isoform X1
uncharacterized protein LOC102723750 isoform X1
uncharacterized protein LOC102723750 isoform X2
uncharacterized protein LOC102723750 isoform X2
uncharacterized protein LOC102723750 isoform X3
uncharacterized protein LOC102724014, partial
uncharacterized protein LOC102724052, partial
uncharacterized protein LOC102724062
uncharacterized protein LOC102724219 isoform X1
uncharacterized protein LOC102724219 isoform X1
uncharacterized protein LOC102724378 isoform X1
uncharacterized protein LOC102724646
uncharacterized protein LOC102724720 isoform X1
uncharacterized protein LOC102724720 isoform X2
uncharacterized protein LOC102724843 isoform X1
uncharacterized protein LOC102724843 isoform X1
uncharacterized protein LOC102724843 isoform X1
uncharacterized protein LOC102724877 isoform X1
uncharacterized protein LOC102724877 isoform X2
uncharacterized protein LOC102724877 isoform X3
uncharacterized protein LOC102724877 isoform X4
uncharacterized protein LOC102724951 isoform X1
uncharacterized protein LOC102724951 isoform X1
uncharacterized protein LOC102724965 isoform X1
uncharacterized protein LOC102724965 isoform X2
uncharacterized protein LOC105369201 isoform X1
uncharacterized protein LOC105369201 isoform X1
uncharacterized protein LOC105369201 isoform X2
uncharacterized protein LOC105369201 isoform X3
uncharacterized protein LOC105369201 isoform X4
uncharacterized protein LOC105369201 isoform X5
uncharacterized protein LOC105369205 isoform X3
uncharacterized protein LOC105369214
uncharacterized protein LOC105369246
uncharacterized protein LOC105369266
uncharacterized protein LOC105369591 isoform X1
uncharacterized protein LOC105369591 isoform X2
uncharacterized protein LOC105369869

uncharacterized protein LOC105369914
uncharacterized protein LOC105370092
uncharacterized protein LOC105370295
uncharacterized protein LOC105370362 isoform X1
uncharacterized protein LOC105370362 isoform X2
uncharacterized protein LOC105370378 isoform X1
uncharacterized protein LOC105370399 isoform X1
uncharacterized protein LOC105370399 isoform X2
uncharacterized protein LOC105370579
uncharacterized protein LOC105370641
uncharacterized protein LOC105370687
uncharacterized protein LOC105370691
uncharacterized protein LOC105370706
uncharacterized protein LOC105370708
uncharacterized protein LOC105370733 isoform X1
uncharacterized protein LOC105370733 isoform X2
uncharacterized protein LOC105370733 isoform X3
uncharacterized protein LOC105370940
uncharacterized protein LOC105370940
uncharacterized protein LOC105370940
uncharacterized protein LOC105371031
uncharacterized protein LOC105371191
uncharacterized protein LOC105371253
uncharacterized protein LOC105371371
uncharacterized protein LOC105371419
uncharacterized protein LOC105371566
uncharacterized protein LOC105371598
uncharacterized protein LOC105371763
uncharacterized protein LOC105371763
uncharacterized protein LOC105371910
uncharacterized protein LOC105371921 isoform X1
uncharacterized protein LOC105371921 isoform X2
uncharacterized protein LOC105371930
uncharacterized protein LOC105371932
uncharacterized protein LOC105371933
uncharacterized protein LOC105371944 isoform X1
uncharacterized protein LOC105371944 isoform X2
uncharacterized protein LOC105372109
uncharacterized protein LOC105372204
uncharacterized protein LOC105372585
uncharacterized protein LOC105372704
uncharacterized protein LOC105372704
uncharacterized protein LOC105372798
uncharacterized protein LOC105372824
uncharacterized protein LOC105372836

uncharacterized protein LOC105372883 isoform X1
uncharacterized protein LOC105372883 isoform X2
uncharacterized protein LOC105373102
uncharacterized protein LOC105373102
uncharacterized protein LOC105373132
uncharacterized protein LOC105373132
uncharacterized protein LOC105373133
uncharacterized protein LOC105373242 isoform X1
uncharacterized protein LOC105373242 isoform X1
uncharacterized protein LOC105373242 isoform X1
uncharacterized protein LOC105373242 isoform X2
uncharacterized protein LOC105373244 isoform X1
uncharacterized protein LOC105373244 isoform X2
uncharacterized protein LOC105373311
uncharacterized protein LOC105373347 isoform X1
uncharacterized protein LOC105373347 isoform X1
uncharacterized protein LOC105373347 isoform X1
uncharacterized protein LOC105373347 isoform X2
uncharacterized protein LOC105373392
uncharacterized protein LOC105373392
uncharacterized protein LOC105373395 isoform X1
uncharacterized protein LOC105373395 isoform X2
uncharacterized protein LOC105373750
uncharacterized protein LOC105373759
uncharacterized protein LOC105373944
uncharacterized protein LOC105374089
uncharacterized protein LOC105374250 isoform 1
uncharacterized protein LOC105374250 isoform X1
uncharacterized protein LOC105374250 isoform X2
uncharacterized protein LOC105374299
uncharacterized protein LOC105374299
uncharacterized protein LOC105374299
uncharacterized protein LOC105374314
uncharacterized protein LOC105374378 isoform X1
uncharacterized protein LOC105374378 isoform X2
uncharacterized protein LOC105374378 isoform X3
uncharacterized protein LOC105374378 isoform X4
uncharacterized protein LOC105374378 isoform X5
uncharacterized protein LOC105374378 isoform X6
uncharacterized protein LOC105374378 isoform X7
uncharacterized protein LOC105374378 isoform X8
uncharacterized protein LOC105374811 isoform X1
uncharacterized protein LOC105374811 isoform X2
uncharacterized protein LOC105375106
uncharacterized protein LOC105375107

uncharacterized protein LOC105375303
uncharacterized protein LOC105375683
uncharacterized protein LOC105375938 isoform X1
uncharacterized protein LOC105375938 isoform X2
uncharacterized protein LOC105376204
uncharacterized protein LOC105376341
uncharacterized protein LOC105376353 isoform X1
uncharacterized protein LOC105376684
uncharacterized protein LOC105376714
uncharacterized protein LOC105376791 isoform X1
uncharacterized protein LOC105376791 isoform X2
uncharacterized protein LOC105376875
uncharacterized protein LOC105376875
uncharacterized protein LOC105376875
uncharacterized protein LOC105376917 isoform X1
uncharacterized protein LOC105377021
uncharacterized protein LOC105377022
uncharacterized protein LOC105377622
uncharacterized protein LOC105377746
uncharacterized protein LOC105377777 isoform X1
uncharacterized protein LOC105377777 isoform X1
uncharacterized protein LOC105377777 isoform X2
uncharacterized protein LOC105378105
uncharacterized protein LOC105378148 isoform X1
uncharacterized protein LOC105378148 isoform X2
uncharacterized protein LOC105378161
uncharacterized protein LOC105378161
uncharacterized protein LOC105378189
uncharacterized protein LOC105378479
uncharacterized protein LOC105378592
uncharacterized protein LOC105378592
uncharacterized protein LOC105379177
uncharacterized protein LOC105379198
uncharacterized protein LOC105379473
uncharacterized protein LOC105379474
uncharacterized protein LOC105379533
uncharacterized protein LOC105379545
uncharacterized protein LOC105379561 isoform X1
uncharacterized protein LOC105379561 isoform X2
uncharacterized protein LOC105379561 isoform X3
uncharacterized protein LOC105379561 isoform X4
uncharacterized protein LOC107983947
uncharacterized protein LOC107983958
uncharacterized protein LOC107983981 isoform X1
uncharacterized protein LOC107983981 isoform X1

uncharacterized protein LOC107983981 isoform X2
uncharacterized protein LOC107983981 isoform X3
uncharacterized protein LOC107983989
uncharacterized protein LOC107983998 isoform X1
uncharacterized protein LOC107983998 isoform X2
uncharacterized protein LOC107984104
uncharacterized protein LOC107984104
uncharacterized protein LOC107984104
uncharacterized protein LOC107984104
uncharacterized protein LOC107984104
uncharacterized protein LOC107984159
uncharacterized protein LOC107984236
uncharacterized protein LOC107984265
uncharacterized protein LOC107984282
uncharacterized protein LOC107984282
uncharacterized protein LOC107984282
uncharacterized protein LOC107984341
uncharacterized protein LOC107984399
uncharacterized protein LOC107984449
uncharacterized protein LOC107984449
uncharacterized protein LOC107984449
uncharacterized protein LOC107984449
uncharacterized protein LOC107984449
uncharacterized protein LOC107984449
uncharacterized protein LOC107984512
uncharacterized protein LOC107984648
uncharacterized protein LOC107984751
uncharacterized protein LOC107984751
uncharacterized protein LOC107984813
uncharacterized protein LOC107984814 isoform X1
uncharacterized protein LOC107984817
uncharacterized protein LOC107984820
uncharacterized protein LOC107984832 isoform X1
uncharacterized protein LOC107984833
uncharacterized protein LOC107984851
uncharacterized protein LOC107984859 isoform X1
uncharacterized protein LOC107984859 isoform X2
uncharacterized protein LOC107984859 isoform X3
uncharacterized protein LOC107984859 isoform X4
uncharacterized protein LOC107984859 isoform X5
uncharacterized protein LOC107984862
uncharacterized protein LOC107984876
uncharacterized protein LOC107985022
uncharacterized protein LOC107985103
uncharacterized protein LOC107985115

uncharacterized protein LOC107985246
uncharacterized protein LOC107985320 isoform X1
uncharacterized protein LOC107985320 isoform X1
uncharacterized protein LOC107985320 isoform X2
uncharacterized protein LOC107985381
uncharacterized protein LOC107985384
uncharacterized protein LOC107985410
uncharacterized protein LOC107985416 isoform X1
uncharacterized protein LOC107985416 isoform X2
uncharacterized protein LOC107985428
uncharacterized protein LOC107985535
uncharacterized protein LOC107985678
uncharacterized protein LOC107985687
uncharacterized protein LOC107985729
uncharacterized protein LOC107985734
uncharacterized protein LOC107985770 isoform X2
uncharacterized protein LOC107985773
uncharacterized protein LOC107985792
uncharacterized protein LOC107985795
uncharacterized protein LOC107985803
uncharacterized protein LOC107985856 isoform X1
uncharacterized protein LOC107985856 isoform X2
uncharacterized protein LOC107985876
uncharacterized protein LOC107985876
uncharacterized protein LOC107985896
uncharacterized protein LOC107985939
uncharacterized protein LOC107985946
uncharacterized protein LOC107985971
uncharacterized protein LOC107986004
uncharacterized protein LOC107986004
uncharacterized protein LOC107986163
uncharacterized protein LOC107986175
uncharacterized protein LOC107986192
uncharacterized protein LOC107986215
uncharacterized protein LOC107986255
uncharacterized protein LOC107986352
uncharacterized protein LOC107986353
uncharacterized protein LOC107986354
uncharacterized protein LOC107986531
uncharacterized protein LOC107986554
uncharacterized protein LOC107986554
uncharacterized protein LOC107986554
uncharacterized protein LOC107986554
uncharacterized protein LOC107986554
uncharacterized protein LOC107986755

uncharacterized protein LOC107986755
uncharacterized protein LOC107986762
uncharacterized protein LOC107986762
uncharacterized protein LOC107986777 isoform X1
uncharacterized protein LOC107986777 isoform X2
uncharacterized protein LOC107986791
uncharacterized protein LOC107986791
uncharacterized protein LOC107986791
uncharacterized protein LOC107986791
uncharacterized protein LOC107986797
uncharacterized protein LOC107986800 isoform X1
uncharacterized protein LOC107986810
uncharacterized protein LOC107986908
uncharacterized protein LOC107986910 isoform X1
uncharacterized protein LOC107986910 isoform X1
uncharacterized protein LOC107986910 isoform X2
uncharacterized protein LOC107987044
uncharacterized protein LOC107987096
uncharacterized protein LOC107987125
uncharacterized protein LOC107987142
uncharacterized protein LOC107987158
uncharacterized protein LOC107987158
uncharacterized protein LOC107987158
uncharacterized protein LOC107987158
uncharacterized protein LOC107987188
uncharacterized protein LOC107987211
uncharacterized protein LOC107987211
uncharacterized protein LOC107987233
uncharacterized protein LOC107987237
uncharacterized protein LOC107987243
uncharacterized protein LOC107987269
uncharacterized protein LOC107987276
uncharacterized protein LOC107987285
uncharacterized protein LOC107987288
uncharacterized protein LOC107987345
uncharacterized protein LOC107987363
uncharacterized protein LOC107987372
uncharacterized protein LOC107987377
uncharacterized protein LOC107987386
uncharacterized protein LOC107987388 isoform X1
uncharacterized protein LOC107987388 isoform X2
uncharacterized protein LOC107987388 isoform X2
uncharacterized protein LOC107987394
uncharacterized protein LOC107987469
uncharacterized protein LOC112267857 isoform X1

uncharacterized protein LOC112267874
uncharacterized protein LOC112267876
uncharacterized protein LOC112267934
uncharacterized protein LOC112267939
uncharacterized protein LOC112267940
uncharacterized protein LOC112267947 isoform X1
uncharacterized protein LOC112267971
uncharacterized protein LOC112267991
uncharacterized protein LOC112268013
uncharacterized protein LOC112268052
uncharacterized protein LOC112268068
uncharacterized protein LOC112268076
uncharacterized protein LOC112268102
uncharacterized protein LOC112268105
uncharacterized protein LOC112268131 isoform X1
uncharacterized protein LOC112268131 isoform X2
uncharacterized protein LOC112268136
uncharacterized protein LOC112268184
uncharacterized protein LOC112268198
uncharacterized protein LOC112268219
uncharacterized protein LOC112268232
uncharacterized protein LOC112268233
uncharacterized protein LOC112268237
uncharacterized protein LOC112268260 isoform X1
uncharacterized protein LOC112268260 isoform X2
uncharacterized protein LOC112268271
uncharacterized protein LOC112268305
uncharacterized protein LOC112268321
uncharacterized protein LOC112268321
uncharacterized protein LOC112268349
uncharacterized protein LOC112268350
uncharacterized protein LOC112268394
uncharacterized protein LOC112268398
uncharacterized protein LOC112268414
uncharacterized protein LOC112268444
uncharacterized protein LOC112268452
uncharacterized protein LOC112268453
uncharacterized protein LOC112268459
uncharacterized protein LOC126661 isoform X1
uncharacterized protein LOC126661 isoform X2
uncharacterized protein LOC285500 isoform X1
uncharacterized protein LOC285500 isoform X2
uncharacterized protein LOC388282
uncharacterized protein LOC388282 isoform X1
uncharacterized protein LOC388780 isoform X1

uncharacterized protein LOC388780 isoform X1
uncharacterized protein LOC388780 isoform X2
uncharacterized protein LOC389199
uncharacterized protein LOC389602
uncharacterized protein LOC389602 isoform X1
uncharacterized protein LOC401040
uncharacterized protein LOC401478 isoform X1
uncharacterized protein LOC401478 isoform X1
uncharacterized protein LOC401478 isoform X2
uncharacterized protein LOC401478 isoform X3
uncharacterized protein LOC401478 isoform X4
uncharacterized protein LOC401478 isoform X5
uncharacterized protein LOC401478 precursor
uncharacterized protein LOC441239
uncharacterized protein LOC642249
uncharacterized protein LOC643365
uncharacterized protein LOC643365 isoform X1
uncharacterized protein LOC644090 isoform X1
uncharacterized protein LOC645188
uncharacterized protein SMIM29 isoform 1 precursor
uncharacterized protein SMIM29 isoform 1 precursor
uncharacterized protein SMIM29 isoform 1 precursor
uncharacterized protein SMIM29 isoform 1 precursor
UPF0488 protein C8orf33 isoform X1
UPF0573 protein C2orf70 isoform 2
UPF0573 protein C2orf70 isoform X5
variable charge X-linked protein 1
variable charge X-linked protein 1 isoform X1
variable charge X-linked protein 3B
variable charge X-linked protein 3
vasohibin-2 isoform X7
vegetative cell wall protein gp1-like
vegetative cell wall protein gp1-like
vegetative cell wall protein gp1-like
vegetative cell wall protein gp1-like
vegetative cell wall protein gp1-like isoform X1
vegetative cell wall protein gp1-like isoform X2
vesicular, overexpressed in cancer, prosurvival protein 1 isoform 11 precursor
vesicular, overexpressed in cancer, prosurvival protein 1 isoform 11 precursor
vesicular, overexpressed in cancer, prosurvival protein 1 isoform 11 precursor
vesicular, overexpressed in cancer, prosurvival protein 1 isoform 5
vesicular, overexpressed in cancer, prosurvival protein 1 isoform 6 precursor
V-set and immunoglobulin domain-containing protein 10-like 2 isoform X3
WAS/WASL-interacting protein family member 2-like
WAS/WASL-interacting protein family member 3-like

WD repeat-containing protein 87 isoform 2
WD repeat-containing protein 87 isoform X1
WNT1-inducible-signaling pathway protein 2 isoform X1
WW domain-containing oxidoreductase isoform X6
XIAP-associated factor 1 isoform X5
xin actin-binding repeat-containing protein 1 isoform 3
zinc finger protein 200 isoform X2
zinc finger protein 268 isoform f
zinc finger protein 74 isoform b
zinc finger protein 778 isoform X4
zinc finger protein 778 isoform X5
zinc finger protein 778 isoform X5
zinc finger protein 778 isoform X5
zinc finger protein 778 isoform X6
ZO-2 associated speckle protein
